# Hidden tellurium speciation resolves the paradox of infinite Te chains in LaCu_*x*_Te_2_

**DOI:** 10.1039/d6sc05234g

**Published:** 2026-08-14

**Authors:** Yihao Wang, Thomas S. Ie, Clayton Halbert, Shima Shahabfar, John Bakir, Hengdi Zhao, Joohee Bang, Justin M. Hoffman, Fatemeh Safari, Paribesh Acharyya, Debattam Sarkar, Adam Balvanz, Anukriti Ghimire, Souvik Sasmal, Christos D. Malliakas, Jesse S. Smith, Nilesh P. Salke, Stephan Rosenkranz, Shanti Deemyad, Chris Wolverton, Russell J. Hemley, Duck Young Chung, Mercouri G. Kanatzidis

**Affiliations:** a Materials Science Division, Argonne National Laboratory Lemont Illinois 60439 USA m-kanatzidis@northwestern.edu; b Department of Chemistry, Northwestern University Evanston Illinois 60208 USA; c Department of Chemistry, University of Illinois Chicago Chicago Illinois 60607 USA; d Department of Materials Science and Engineering, Northwestern University Evanston Illinois 60208 USA; e Department of Physics and Astronomy, University of Utah Salt Lake City Utah 84112 USA; f X-ray Science Division, Argonne National Laboratory Lemont Illinois 60439 USA; g Department of Physics, University of Illinois Chicago Chicago Illinois 60607 USA; h Department of Earth and Environmental Sciences, University of Illinois Chicago Chicago Illinois 60607 USA

## Abstract

Low-dimensional polytellurides are of broad interest because their flexible bonding gives rise to unusual electronic states, structural instabilities, and transport phenomena, yet their average crystal structures can obscure the local tellurium motifs that actually control these properties. The electronic character of LnCu_*x*_Te_2_ (Ln = lanthanide) remains unresolved because electronic structure calculations based on the average crystal structure predict metallic behavior, whereas experiments consistently show semiconducting transport. Here we examine this discrepancy in LaCu_*x*_Te_2_ by combining low-temperature single-crystal X-ray diffraction, total-scattering analysis, electrical transport measurements, high-pressure studies, and first-principles calculations. Single-crystal diffraction at 100 K reveals an incommensurately modulated structure that is absent at room temperature, and X-ray total scattering indicates local unit-cell tripling along the direction of the apparent linear Te chains. These results show that the crystallographic infinite chain is not a real chemical entity but an average motif that masks locally distorted Te arrangements. In this picture, the anomalous transport of LaCu_*x*_Te_2_ is linked to chain distortion and structural disorder rather than to an idealized delocalized Te chain electronic state. Consistent with this interpretation, LaCu_*x*_Te_2_ exhibits semiconducting behavior over the full temperature range studied, variable-range hopping at low temperature, and quasi-linear nonsaturating magnetoresistance attributable to spatially inhomogeneous carrier mobility. Under pressure, the room-temperature structure remains unchanged and the charge transport activation energy decreases, yet metallic behavior does not emerge. Together, these results reconcile the apparent conflict between ideal-chain calculations and experiment, demonstrating that hidden local distortion within an average one-dimensional tellurium motif governs the electronic behavior of LaCu_*x*_Te_2_.

## Abstract 2

Instead of infinite tellurium chains, LaCu_*x*_Te_2_ intrinsically forms a statistically disordered collinear arrangement of monotelluride and oligotelluride species, yielding semiconducting transport and quasi-linear magnetoresistance at low temperatures.

## Graphical abstract

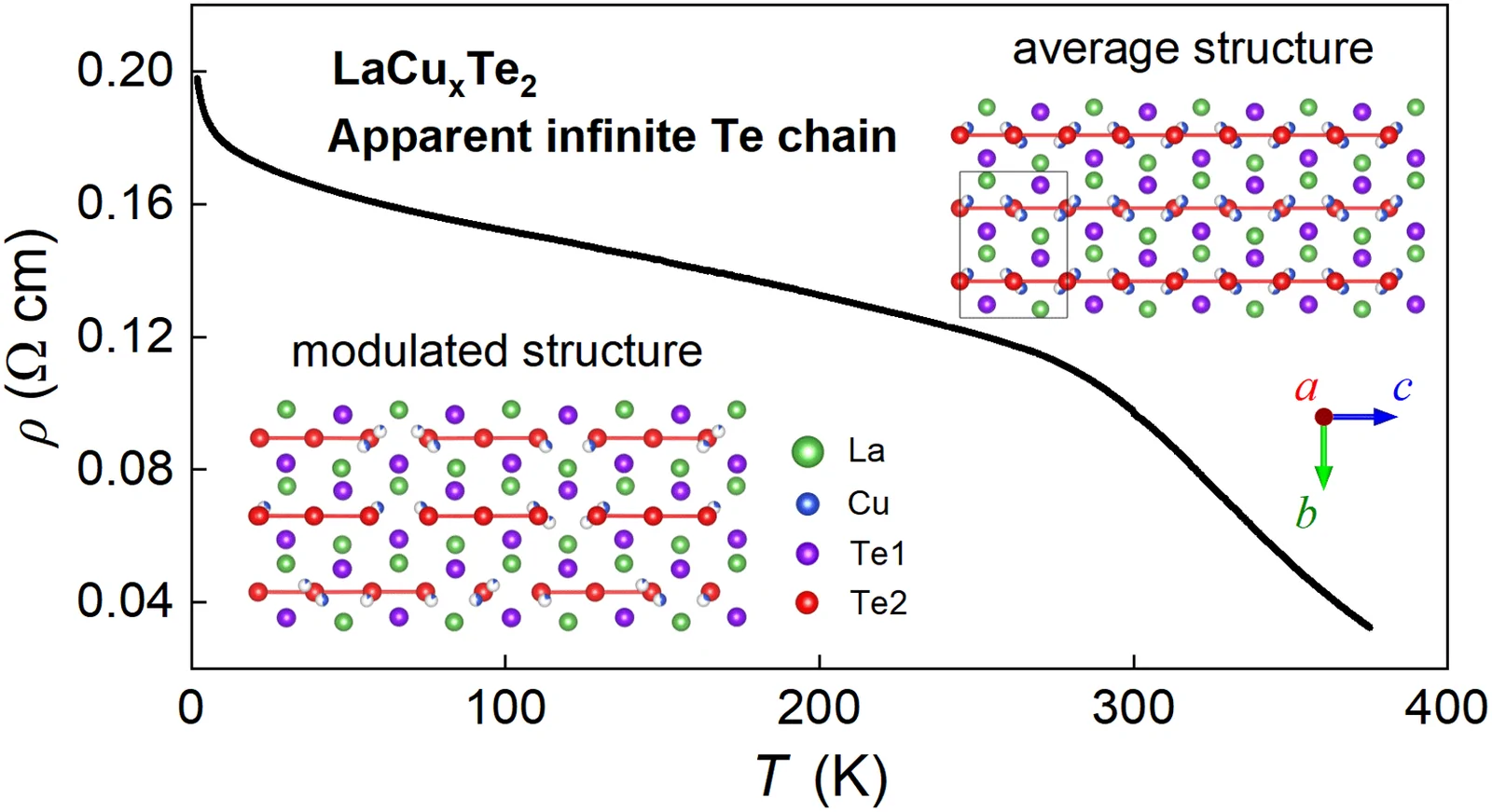

## Introduction

Polytellurides are of broad interest because the unusual bonding flexibility of tellurium gives rise to diverse anionic motifs, low-dimensional structures, and unusual electronic and lattice behaviors. In such materials, relatively small changes in Te–Te connectivity can strongly reshape electronic structure and transport, making them valuable platforms for exploring how local bonding motifs govern physical properties. Owing to the hypervalent character of its 5p electrons, tellurium forms a wide range of structural motifs, ranging from zero-dimensional oligomers to three-dimensional covalent networks.^1–5^ Among polytellurides containing Te_*n*_^2−^ anions, bent geometries are most common: Te_3_^2−^ trimers typically adopt Te–Te–Te angles of 90–105°, whereas Te_4_^2−^ tetramers and longer units usually form zigzag chains. By contrast, oligomeric motifs (*n* > 2) with linearly arranged tellurium atoms are relatively rare. The nature of the Te–Te interaction also depends strongly on bond distance, with separations of 2.7–2.9 Å corresponding to covalent bonding and distances greater than 3.5 Å indicating predominantly van der Waals interactions. This bonding versatility gives tellurides a wide range of electronic characters, from semiconducting to metallic, and can produce unconventional physical properties. For example, single-crystalline trigonal tellurium exhibits narrow-gap semiconducting behavior and topological edge states associated with helical, not linear Te chains and interchain electronic delocalization.^6–8^ In transition-metal dichalcogenides and trichalcogenides, instabilities of chalcogen nets can give rise to charge-density waves.^1,9–11^ These features make polytellurides especially attractive systems for uncovering unusual structure–property relationships, particularly in cases where the chemically meaningful local Te arrangement may differ from the motif suggested by the average crystal structure.

Dimensionality provides another important lever for controlling physical properties. In low-dimensional materials, coupling between electronic structure and the lattice often becomes especially pronounced, increasing the tendency toward structural distortion and electronically driven instabilities. A canonical example is the charge-density wave, in which Fermi-surface nesting or electron-phonon coupling produces a periodic modulation of charge density accompanied by lattice distortion.^3,12–16^ Reduced dimensionality can also narrow bandwidths, enhance correlation effects, and promote unusual transport and collective behavior.^17–19^ Because such states are often highly sensitive to external stimuli, including pressure and photoexcitation, low-dimensional materials serve as valuable platforms for probing the interplay between bonding, structure, and electronic response.^20,21^

Within this broader context, LnCu_*x*_Te_2_ (Ln = lanthanide) compounds present a long-standing structural and electronic paradox.^22–29^ Their average crystal structures suggest infinite linear tellurium chains, with Te–Te separations of about 3.1 Å, intermediate between typical covalent and van der Waals interactions. Electronic structure calculations based on the average crystallographic structure predicted delocalized states and metallic behavior, whereas transport measurements consistently show semiconducting behavior.^24^ This discrepancy suggests that the average structure may not capture the chemically and electronically relevant local arrangement. Given the low-dimensional character of these materials, structural modulation of the Te sublattice is a natural possibility. By including distortions of the Cu and Te sublattices, semiconducting electronic structures were theoretically reproduced.^24,27,29^ Although these proposed distortion modes differ from the incommensurate modulation observed experimentally in the present work, they demonstrate that the calculated electronic behavior is highly sensitive to the assumed local structure. More importantly, in contrast to the multiple theoretical studies of LnCu_*x*_Te_2_, direct experimental evidence for such distortions has remained elusive.^24,28,29^ This absence may further reflect the averaging inherent in room-temperature diffraction measurements, as well as the air sensitivity of tellurides, which can alter both structure and physical properties.^23,30^ Together, these observations point to unresolved local structural complexity as a possible origin of the mismatch between calculation and experiment and provide the central motivation for the present study.

In this work, we address this paradox by combining low-temperature single-crystal X-ray diffraction, total-scattering analysis, electrical transport measurements, high-pressure studies, and first-principles calculations on LaCu_*x*_Te_2_. Low-temperature diffraction reveals an incommensurately modulated structure, while total X-ray scattering provides evidence for unit-cell tripling along the apparent tellurium-chain direction. These results show that the crystallographic linear chain is an average motif that conceals a more complex local Te arrangement. Based on charge-balance principles, the most plausible model is that the apparent Te chains in LaCu_*x*_Te_2_ comprise Te^2−^ centers and linear [Te_3_]^4−^ units, whose local connectivity is averaged in the diffraction experiment into an apparently continuous one-dimensional motif. Consistent with this structural picture, LaCu_*x*_Te_2_ exhibits variable-range hopping at low temperature and quasi-linear magnetoresistance, indicating that the modulation strongly influences carrier transport and creates an electronically inhomogeneous landscape. Under pressure, the room-temperature crystal structure remains unchanged and the activation energy decreases, yet metallic behavior does not emerge. Our findings provide a structurally grounded explanation for the semiconducting behavior of LaCu_*x*_Te_2_ and show that its incommensurately modulated state differs fundamentally from a conventional charge-density-wave ground state.

## Results and discussion

### Average structure

**Fig. 1:**
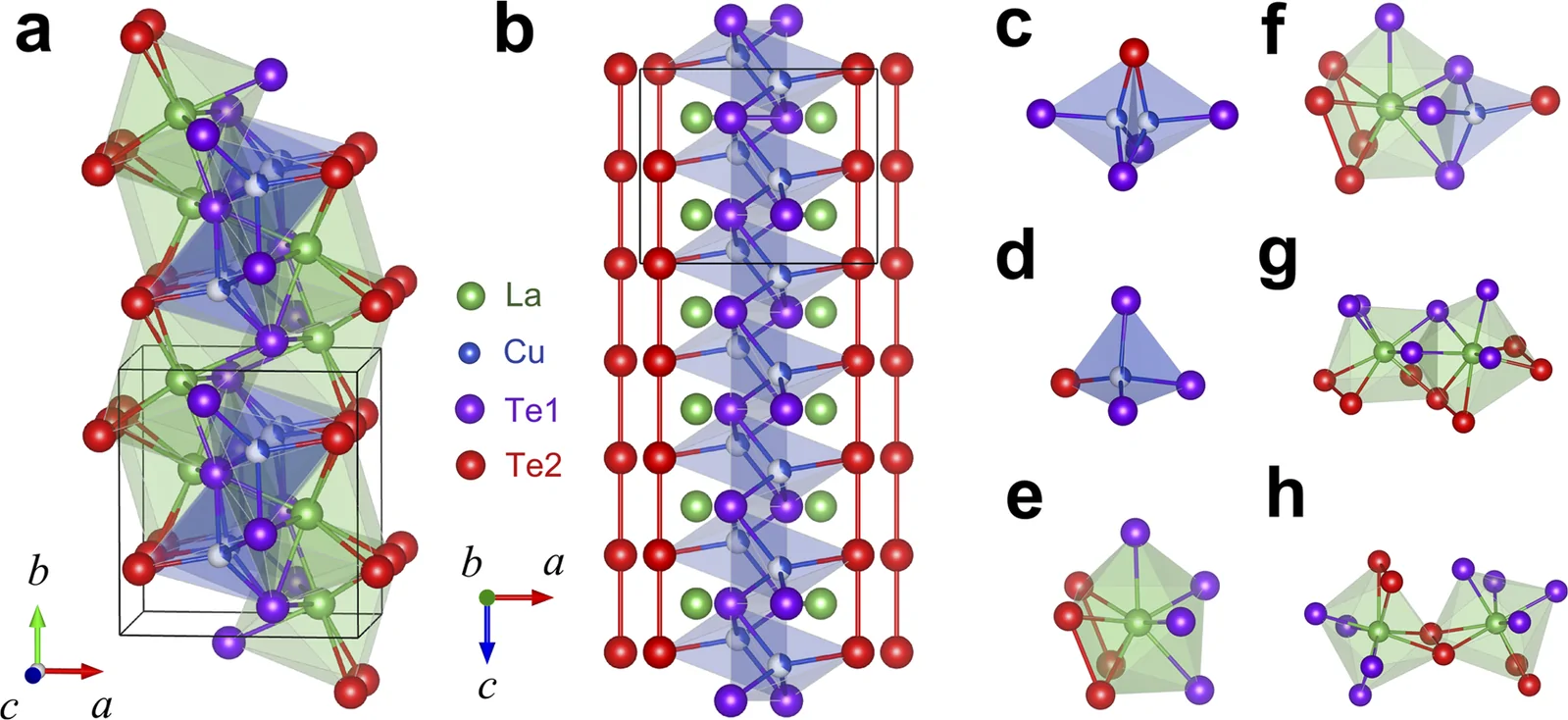
(a) Average structure of LaCu_*x*_Te_2_ at room temperature. (b) Average structure of LaCu_*x*_Te_2_ viewed along the *b*-axis. (c) An unreasonable face-sharing [CuTe_4_] motif with Cu–Cu distances of 1.2 Å. (d) and (e) Building blocks showing the coordination environments of La and Cu in LaCu_*x*_Te_2_. (f) Face-sharing connectivity between [LaTe_8_] and [CuTe_4_]. (g) Face-sharing connectivity between [LaTe_8_]. (h) Edge-sharing connectivity between [LaTe_8_].

We begin by establishing the room-temperature average structure of LaCu_*x*_Te_2_, which provides the structural baseline for evaluating the origin of its unusual electronic behavior. The original study by Ibers and coworkers described LnCu_*x*_Te_2_ as an orthorhombic *Pbcm* structure containing partially occupied Cu sites and infinite linear Te chains, and this average structure was used to rationalize the metallic behavior of these compounds.^24^ Our room-temperature single-crystal X-ray diffraction (SXRD) refinement reproduces this average structural model for LaCu_*x*_Te_2_, including the apparent one-dimensional Te chain motif. According to the refinement results (Table 1 and Table S1), LaCu_*x*_Te_2_ crystallizes in the orthorhombic space group *Pbcm* (No. 57) at room temperature (Fig. 1a and b). The La atoms occupy symmetry-equivalent positions and are eight-coordinate with Te, forming [LaTe_8_] polyhedra, a common coordination environment in La–Te-containing compounds.^31–33^ The Te atoms are divided into two crystallographically distinct sites. The Te1 atoms occupy the 4d Wyckoff position with site symmetry *m* and correspond to discrete Te^2−^ anions, whereas the Te2 atoms occupy the 4c Wyckoff position with twofold rotational symmetry. The Te2 atoms form the apparent infinite one-dimensional linear chains along the *c*-axis, with Te2⋯Te2 separations of approximately 3.16 Å, a value intermediate between typical covalent Te–Te bonds and van der Waals contacts. Thus, at the level of the room-temperature average structure, our refinement confirms the essential Te-chain motif reported by Ibers and co-workers. In the previous study, Te2 was found to contribute significantly to the total density of states near the Fermi level, suggesting metallic behavior if the apparent infinite Te chains of the average structure are taken at face value.^24^

**Table 1:** Crystallographic data and structure refinement for X-ray diffraction measurements on single-crystalline LaCu_*x*_Te_2_ at room temperature and 100 K

Phase labels	LaCu_0.41_Te_2_ RT	LaCu_0.347_Te_2_ LT
Formula weight (g mol^−1^)	420.16	416.2
Temperature (K)	293.16	100.03
Radiation source	Mo Kα, *λ* = 0.71073 Å	Mo Kα, *λ* = 0.71073 Å
Crystal system	Orthorhombic	Monoclinic
Space group	*Pbcm*	*P*2_1_/*m(*0*b*½*)s*0
*a* (Å)	7.7110(15)	7.69960(10)
*b* (Å)	8.5874(17)	6.27810(10)
*c* (Å)	6.3106(13)	8.58650(10)
*α* (^o^)	90	90
*β* (^o^)	90	90.0946(13)
*γ* (^o^)	90	90
Modulated wavevector	N/A	*q* = 0.371170b* + 0.500000c*
Volume (Å^3^)	417.87(14)	415.061(10)
Calculated density (g cm^−3^)	6.676	6.6597
Absorption coefficient (mm^−1^)	25.683	25.555
*R*_1_ (*I* > 2*σ*(*I*))^a^	0.0315	0.0475
*R*_1_ (all data)	0.0337	0.1588
w*R*_2_ (*I* > 2*σ*(*I*))^b^	0.0843	0.0485
w*R*_2_ (all data)	0.0853	0.1594
*R*_1_ 1st order satellites (*I* > 2*σ*(*I*))	N/A	0.0495
w*R*_2_ 1st order satellites (*I* > 2*σ*(*I*))	N/A	0.1370
*R*_1_ 1st order satellites (all data)	N/A	0.0539
w*R*_2_ 1st order satellites (all data)	N/A	0.1384
*T*_min_ and *T*_max_ coefficients	N/A	0.348 and 0.368
Largest peak/deepest hole (e^−^/Å^3^)	3.06/−1.55	6.34/−3.19

a *R*_1_ = ∑‖*F*_o_| − |*F*_c_‖/∑|*F*_o_|.

b
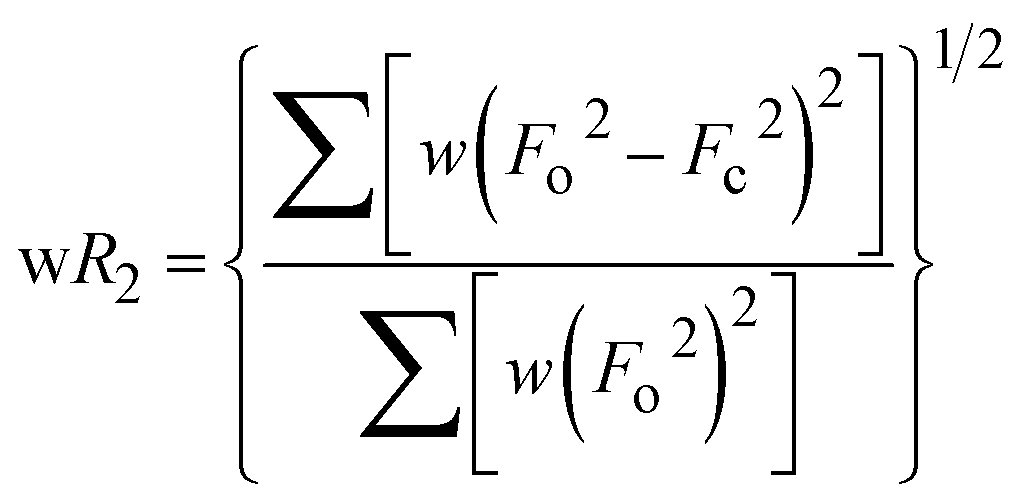
; where *w* = 1/[*σ*^2^*F*_o_^2^ + (*AP*)^2^ + *BP*], and 
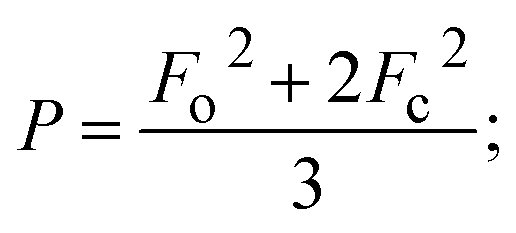
*A* and *B* are the weight coefficients.

As shown in Fig. 1c, Cu is coordinated by Te in distorted [CuTe_4_] tetrahedra, a common motif in compounds containing both Cu and Te.^34–37^ If the Cu site were fully occupied in LaCu_*x*_Te_2_, face-sharing [CuTe_4_] tetrahedra would place neighboring Cu atoms only about 1.19 Å apart, which is not chemically reasonable. The Cu site must therefore be only partially occupied, consistent with experimental results.^22^ We exclude the unreasonable Cu arrangement and plot the crystal structures as shown in Fig. 1a and b. Corresponding to the partially occupied Cu, the anisotropic parameter *U*_33_ of the Cu site is twice the value along the other directions (Table S2), indicating a preferred thermal vibration direction along the *c*-axis at room temperature. As shown in Fig. 1f, the connectivity between [LaTe_8_] and [CuTe_4_] always forms face-sharing configurations. In contrast, [LaTe_8_] polyhedra interconnect with each other by sharing either face or edge (Fig. 1g and h), among which the latter mode, *i.e.*, edge-sharing connectivity, indicates a unique configuration among reported compounds.

In the hypothetical fully occupied composition LaCuTe_2_, simple charge balance would assign La^3+^, Cu^+^, and discrete Te^2−^ anions, with no requirement for Te–Te bonding. In the known LaCu_*x*_Te_2_ compounds, however, *x* is less than 1 and typically falls in the range 0.26–0.60,^22^ requiring partial oxidation of the tellurium sublattice. This electron count is most naturally accommodated by local Te–Te bonding and the formation of polytelluride fragments, Te_*n*_^2−^, whose distribution depends on the exact Cu content. More unusually, it could also be represented by a uniform infinite Te chain with fractional Te–Te bonding distributed along the chain. The latter would be a special bonding situation, but it cannot be excluded from the room-temperature average structure alone. This ambiguity defines the central paradox addressed in this work: the room-temperature average structure reproduces the previously reported infinite Te chain model, whereas the Cu deficiency and electron count require partial oxidation and chemical differentiation of the tellurium sublattice. The low-temperature crystallographic analysis described below resolves this discrepancy by revealing how the Te–Te bonding is actually organized. This situation can give rise to a mixture of polytelluride species together with structural modulation and disorder at the Te sites. Detailed discussion of these possibilities is presented in the following sections. Because LaCu_*x*_Te_2_ exists over a Cu-deficient compositional range, the exact value of *x* differs somewhat among crystals, powder samples, and computational models used in this study. The central point, however, is independent of this variation: all experimentally relevant compositions are strongly Cu-deficient and therefore require partial oxidation and chemical differentiation of the Te sublattice. The variable Cu content reported for LaCu_*x*_Te_2_, together with the different Cu occupancies refined for crystals examined in this work, indicates that this structure accommodates a finite compositional width with respect to copper. This phase width is chemically important because *x* directly determines the average oxidation state of the tellurium sublattice. As the Cu content varies from crystal to crystal or from reaction to reaction, the relative populations of isolated Te^2−^ centers and oligotelluride fragments are expected to change accordingly. Additional crystallographic information for the room-temperature structure, including atomic coordinates, equivalent isotropic displacement parameters, anisotropic displacement parameters, bond lengths, bond angles, and site occupancies, is provided in Tables S1–S5.

### Band structure calculations

Having established that the room-temperature average structure reproduces the previously reported apparent infinite Te chain motif, we next examined the electronic consequences of this structural model using density functional theory (DFT) calculations. These calculations are not intended to resolve the low-temperature modulated structure, but rather to evaluate the electronic behavior predicted when the apparent infinite Te chain of the average structural model is taken at face value.

**Fig. 2:**
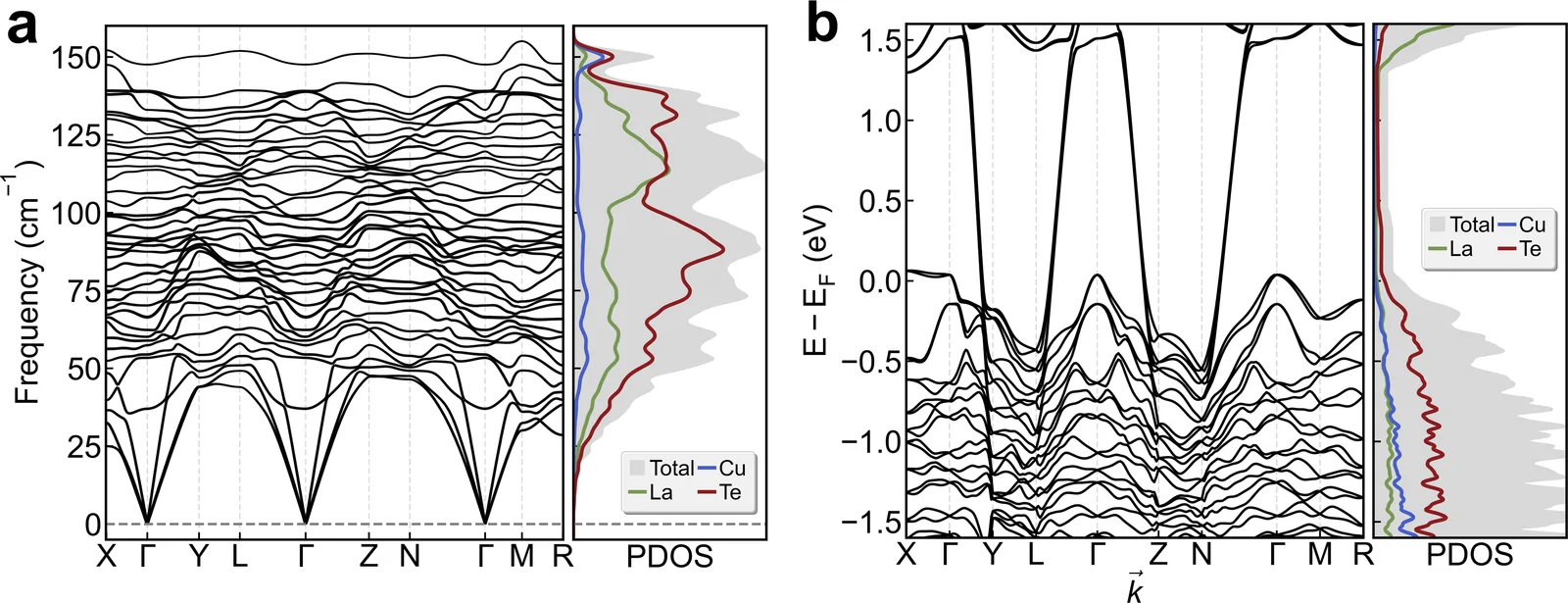
(a) Calculated phonon dispersion and phonon DOS of La_4_CuTe_8_ (*x* = 0.25). (b) Calculated electronic band structure and partial DOS of La_4_CuTe_8_ (*x* = 0.25).

Formation-energy calculations were first used to assess the relative stability of La–Cu–Te compositions considered in this work. As shown in Table S6, La_3_CuTe_5_ has the lowest formation energy and is the most stable phase among the La–Cu–Te compositions examined.^33^ For LaCu_*x*_Te_2_, the calculated formation energy is higher but remains negative, consistent with its experimental accessibility. To evaluate the electronic structure of the Te-chain-containing average model, we performed DFT calculations on ordered LaCu_0.25_Te_2_, represented as La_4_CuTe_8_. This composition lies only 14 meV atom^−1^ above the convex hull (Table S6), indicating a slightly metastable phase that can plausibly be stabilized under finite-temperature synthetic conditions. The calculated phonon dispersion and phonon density of states for LaCu_0.25_Te_2_ are shown in Fig. 2a. The absence of imaginary phonon modes indicates that this ordered model is dynamically stable within the harmonic approximation.

Consistent with the previous report, the average-structure model containing uniform infinite Te chains yields a metallic electronic structure (Fig. 2b).^24^ The bands crossing the Fermi level are dominated by highly dispersive Te p states, showing that the apparent infinite Te chain is not a passive structural feature but directly controls the calculated metallicity. The large bandwidth reflects strong orbital overlap within the tellurium sublattice, a feature often associated with high conductivity in low-dimensional chalcogenides.^38^ We also find that the dynamical stability of the LaCu_*x*_Te_2_ model depends sensitively on Cu occupancy. For *x* = 0.5, pronounced imaginary phonon modes appear in the phonon dispersion (Fig. S1), indicating a tendency toward structural distortion or modulation. Thus, the electronic-structure calculations sharpen the central problem: the same average structural model that reproduces the previously reported infinite Te chain motif also predicts metallic behavior through dispersive Te p bands at the Fermi level. If this uniform-chain model describes the true electronic structure of LaCu_*x*_Te_2_, metallic transport should be observed. This prediction is directly tested by the transport measurements discussed below.

In LaCu_*x*_Te_2_, the structural modulation gives rise to alternating Te–Te distances along the linear tellurium chains. Similar bond distance distributions have been reported in several chalcogen compounds, where multicenter or hypervalent interactions may appear.^39,40^ Further electronic-structure analysis is needed to clarify the nature of these interactions. Calculations based on a model that incorporates the experimentally observed distortion may provide further insight into the relationship between the structural distortion and the electronic structure. However, since both studies require substantial calculations, *e.g.*, constructing a supercell or generating a special quasi-random structure (SQS) to deal with the partially occupied Cu, we therefore regard them as separate studies beyond the scope of present experimental and structural investigations.

### Modulated structure

**Fig. 3:**
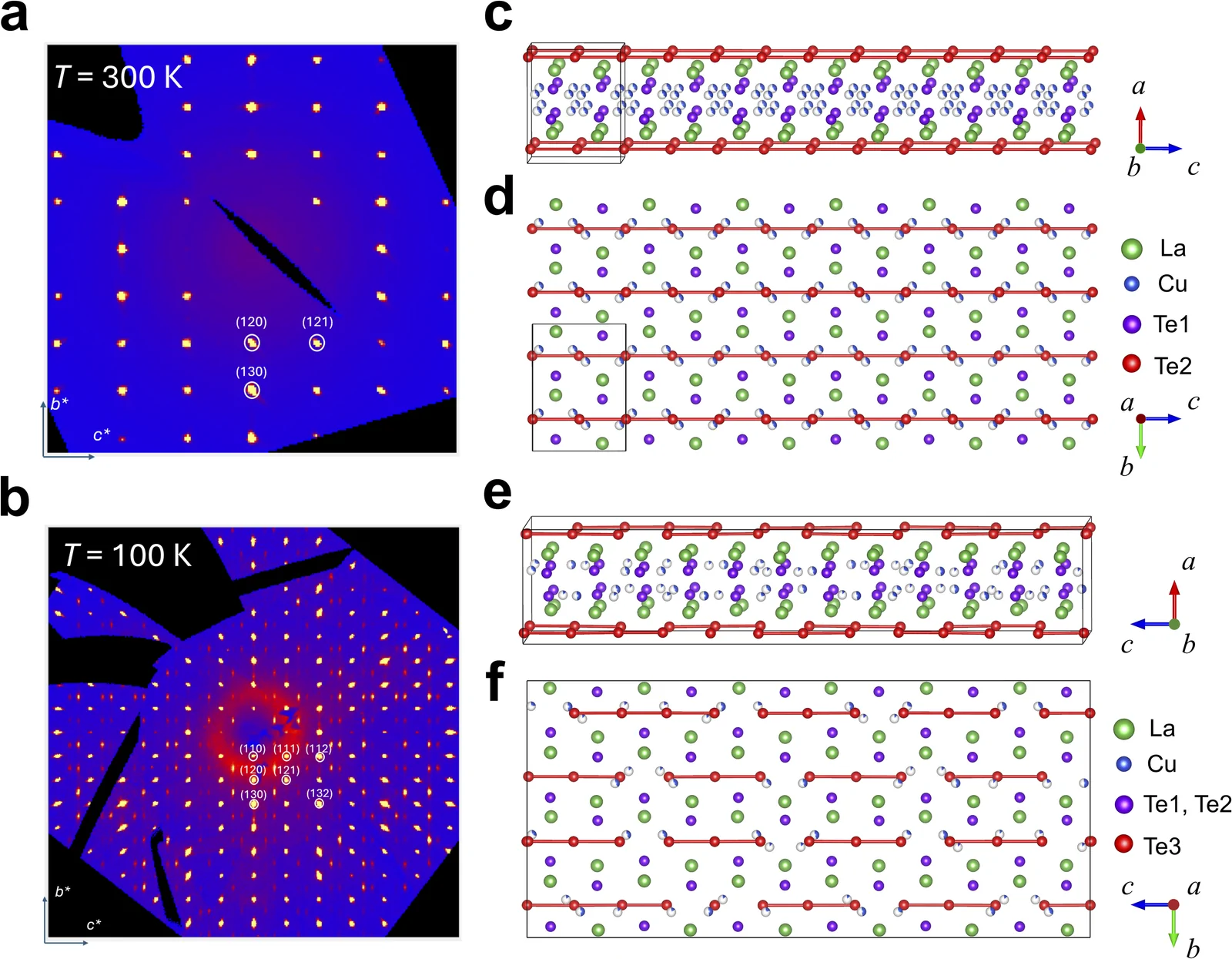
(a) and (b) Diffraction patterns of a LaCu_*x*_Te_2_ single crystal in the reciprocal *bc*-plane at 300 K and 100 K, respectively. (c) and (d) Side view along *b*-axis and top view along the *a*-axis of the LaCu_*x*_Te_2_ crystal structure at 300 K. (e) and (f) Side view along *b-*axis and top view along *a*-axis of the LaCu_*x*_Te_2_ crystal structure at 100 K for an approximated 2*b* × 6*c* expansion of the incommensurate cell. Here the *b* and *c* axes are interchanged relative to the original cell from the structure refinement (Table 1).

The band-structure calculations described above show that the room-temperature average structure, if interpreted as containing uniform infinite Te chains, should give metallic behavior. This prediction raises a direct structural question: are the apparent Te chains truly uniform, or does the room-temperature average structure conceal a lower-temperature modulation that differentiates the Te–Te contacts? As discussed in the Introduction, one-dimensional Te chains are susceptible to structural distortions, especially when partial oxidation produces fractional Te–Te bonding. To search for such distortions in LaCu_*x*_Te_2_, we carried out single-crystal X-ray diffraction measurements at 100 K. To facilitate comparison with the room-temperature structure, we use the crystallographic setting in which the *b* and *c* axes of the monoclinic subcell are interchanged relative to the original cell from the structure refinement (Table 1). Clear satellite reflections appear in the (100) reciprocal-space section at 100 K (Fig. 3b), whereas no corresponding reflections are observed in the room-temperature diffraction pattern (Fig. 3a). The modulation wavevector is indexed as *q* = 0.500000b* + 0.371170c*, indicating an incommensurately modulated structure. The incommensurate component of approximately 0.37 lies along the original *c*-axis, which corresponds to the Te chain direction, immediately suggesting that the modulation is closely associated with the apparent one-dimensional Te chains.

For systems with a single incommensurate modulation, including structural, charge-density wave, or magnetic modulations, a (3 + 1)-dimensional superspace approach is generally used to describe the long-range order.^41–43^ A conventional displacement-modulation model was applied for the refinement:^44,45^

1 *r*(*t*) = *r*_0_ + *A* sin(2π*t*) + *B* cos(2π*t*)

with *t* = *q*·(*R* + *r*_0_). Here, *A* and *B* denote the modulation amplitudes, *r*_0_, *q*, and *R* represent the average position, incommensurate modulation vector, and lattice translation vector, respectively. Based on the refinements, the structural modulation in LaCu_*x*_Te_2_ results in a change of the space group from an orthorhombic cell with the space group, *Pbcm*, into a monoclinic subcell with the superspace group, *P*2_1_/*m(*0*b*½*)s*0. The crystallographic data and structure refinement information are exhibited in Tables 1 and S7–S11 in the SI.

The resulting modulated structure is shown in Fig. 3c–f. Most importantly, the single Te2⋯Te2 separation of approximately 3.16 Å in the room-temperature average structure is resolved at 100 K into a distribution ranging from 2.98 to 3.28 Å. Thus, the apparent uniform infinite Te chain of the average structure is retained only as an average motif; at low temperature, the Te sublattice is modulated into alternating shorter and longer Te–Te contacts. This finding addresses the structural part of the paradox: the infinite Te chain motif reported previously and reproduced in our room-temperature refinement is real at the average-structure level, but it does not correspond to a uniform Te–Te bonding pattern in the modulated structure.

In addition to modulation of the Te chains, the refinement also reveals rearrangement of the partially occupied Cu sites. This point is important because different types of incommensurate modulation can have different microscopic origins. In band-driven systems, such as conventional Peierls or charge-density-wave materials, the dominant distortion is often concentrated within the low-dimensional electronic substructure, while the remaining atoms undergo only smaller secondary displacements. By contrast, lattice-driven instabilities can involve more collective distortions of the framework, as commonly observed in nickelates and perovskite oxides.^46–48^ In LaCu_*x*_Te_2_, the coupled displacement of both Te and Cu suggests that the modulation is not simply a distortion of an isolated Te chain, but instead reflects a broader lattice instability involving the Te sublattice and the partially occupied Cu network. This interpretation is consistent with the phonon calculations, where imaginary modes appear for higher Cu occupancy, indicating an intrinsic tendency toward structural modulation. The absence of imaginary modes in this particular ordered LaCu_0.25_Te_2_ model does not exclude modulation in the real material, where Cu occupational disorder, finite-temperature effects, and compositions closer to *x* ≈ 0.26–0.6 can alter the lattice instability landscape.

### Modulation of the Te chains

**Fig. 4:**
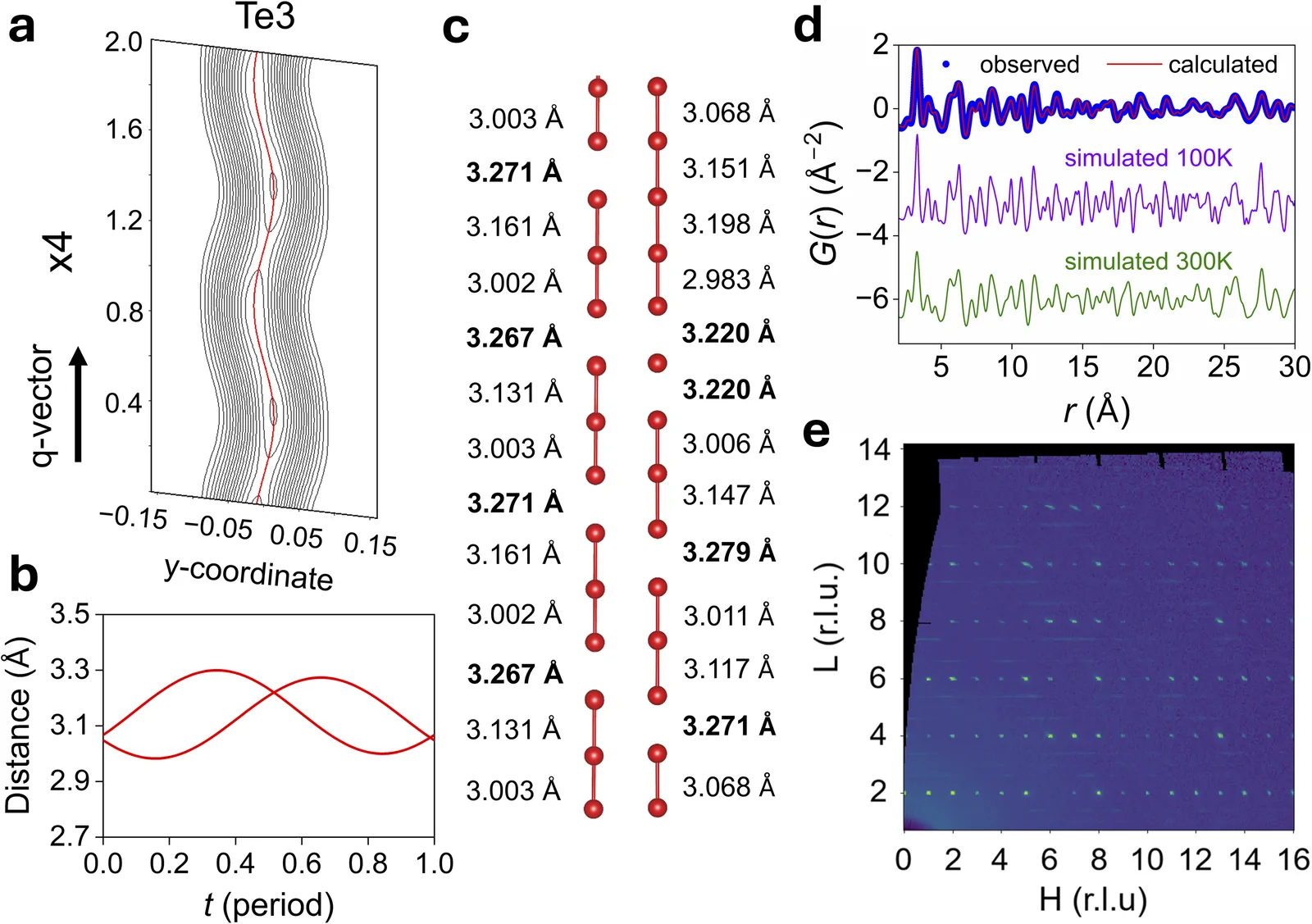
(a) Two-dimensional electron density map which reflects the atomic displacement of Te along the *q*-vector as a function of *y*-coordinate resulting in the distorted chain. (b) Te3–Te3 bond distance within the chain as a function of the modulated phase *t*. The two curves here reflect the modulation mode of the two chains in 300 K unit cell. (c) The bond distances between Te and Te in the Te chains upon simulating a 2*b* × 3*c* (left) and a 2*b* × 6*c* (right) expansion of the subcell at 100 K. (d) X-ray powder PDF data collected at 300 K (bold line) and its comparison with the simulated data at 100 K and 300 K. The red solid line indicates the refinement result based on the 300 K structure. (e) Total X-ray scattering reflection pattern on the *h*0*l* plane collected at 30 K.

To understand how the incommensurate modulation changes the apparent Te chain motif, we analyzed the positional modulation of the Te atoms within the chain. The Te atoms were refined using a positional modulation wave (Fig. 4a), which describes the displacement of Te along the *y* coordinate as a function of the modulation phase. This displacement converts the single Te–Te separation observed in the room-temperature average structure into a distribution of Te–Te distances within the chain. Plotting the Te–Te distances as a function of the modulation phase reveals that the two Te–Te contacts vary in a complementary sinusoidal manner (Fig. 4b). As one Te–Te contact shortens, the adjacent contact lengthens, producing an alternating distribution of shorter and longer Te–Te distances instead of a uniform linear chain. An approximate visualization of this distortion using an expanded subcell is shown in Fig. 4c. When a 3.2 Å cutoff is applied to define Te–Te bonding, the apparent infinite chain breaks into a distribution of finite linear Te fragments, with Te3 units appearing as a prominent motif.

We next examined whether this local modulation could be detected by pair distribution function (PDF) analysis. The purity of polycrystalline LaCu_0.5_Te_2_ was verified by performing Rietveld refinements on the powder X-ray diffraction patterns (Fig. S2). Room-temperature powder PDF data for LaCu_0.5_Te_2_ collected at Sector 11 of the Advanced Photon Source can be fitted well using the orthorhombic *Pbcm* average structure (Fig. 4d), indicating that the average local coordination environment is broadly captured by the room-temperature model. However, the Te2–Te2 distance of approximately 3.15 Å overlaps strongly with the La–Te distance near 3.31 Å, making the Te chain distortion difficult to isolate in the powder PDF. Peak-decomposition analysis shows that La–Te correlations contribute approximately five times more strongly to the total *G*(*r*) than the Te2–Te2 correlations (Fig. S3). In addition, simulated PDF curves based on the 300 K and 100 K structures show only negligible differences (Fig. 4d). Thus, conventional powder PDF analysis is not sufficiently sensitive to resolve the detailed Te chain modulation in LaCu_*x*_Te_2_.

To overcome this limitation, we performed total X-ray scattering on a LaCu_*x*_Te_2_ single crystal. In general, the morphology of diffuse scattering directly reflects the underlying structural correlation lengths: sharper features correspond to long-range correlations, while broader diffuse features indicate short-range order. Furthermore, compared with powder PDF, total X-ray scattering preserves orientational information and is therefore better suited for identifying correlations along a specific crystallographic direction. As shown in Fig. 4e and S4, discontinuous diffuse scattering streaks appear at 30 K and persist up to 265 K. Along the *l* direction, the superstructure reflections appear at one-third-integer positions, indicating a unit-cell tripling along the Te chain direction. In contrast, the same reflections are significantly elongated along *h* and *k*, revealing short lateral correlation length. Based on the anisotropic diffuse scattering features, the superstructure is well-ordered along the *c*-axis, whereas neighboring superstructural domains are weakly correlated or uncorrelated in the *ab* plane.

The combined superstructure refinement and total X-ray scattering therefore show that the apparent infinite Te chain in the room-temperature average structure is not a chemically uniform chain of equivalent Te atoms. Instead, the Te sublattice is modulated into an arrangement containing shorter and longer Te–Te contacts, with local correlations consistent with finite oligotelluride fragments, including prominent trimer-like units. This interpretation also resolves the charge-balance problem. With La treated as La^3+^ and Cu as Cu^+^, Cu deficiency requires partial oxidation of the tellurium sublattice. At the level of the room-temperature average structure, this partial oxidation could appear as a uniform infinite Te chain with fractional Te–Te bonding distributed along the chain. The low-temperature modulation shows instead that the bonding is spatially differentiated: the average chain is more naturally understood as a crystallographic average of locally distinct tellurium environments, including isolated telluride ions and linear oligotelluride fragments.

Thus, the structural paradox is resolved at the local and modulated-structure levels. The infinite linear Te chain reported previously and reproduced in our room-temperature refinement is a valid average structural motif, but it does not represent a uniform bonding pattern. Rather, the average chain emerges from a modulated and partially disordered tellurium sublattice in which Te–Te bonding is concentrated into finite fragments. This distinction is critical for interpreting the electrical behavior: the uniform-chain average model predicts metallicity, whereas the modulated structure disrupts the continuous Te p-orbital pathway that would otherwise support metallic transport.

Having resolved the modulated structure and local order, we can now revisit the charge balance and local tellurium speciation in LaCu_*x*_Te_2_. From this standpoint, the average formulation LaCu_*x*_Te_2_ is difficult to reconcile with a genuine infinite linear [Te]_*n*_ chain built from uniformly equivalent tellurium atoms. With La most reasonably treated as La^3+^ and Cu as Cu^+^, the Cu deficiency requires partial oxidation of the tellurium sublattice relative to a lattice composed only of isolated Te^2−^ anions. The combination of stoichiometry and refined Te–Te distances is therefore more naturally explained by a mixture of isolated telluride ions and oligotelluride fragments than by a chemically uniform infinite chain. The average linear “chain” seen crystallographically is better understood as a statistically disordered, collinear arrangement of distinct tellurium species, most plausibly combinations of Te^2−^ and tritelluride units such as linear [Te_3_]^4−^, whose local connectivity is averaged in the diffraction experiment into an apparently continuous one-dimensional motif. We note that these [Te_3_]^4−^ ions are isoelectronic with the better known linear [I_3_]^−^^49,50^ and [Sb_3_]^7−^ ions^51–54^ and are formed by the addition of Te^2−^ to [Te_2_]^2−^ species. This interpretation is consistent with the room-temperature average structure, in which the Te2–Te2 separations are intermediate at about 3.16 Å, and with the low-temperature modulation, where those distances spread over a broad range and the chain breaks into fragments when a reasonable bonding cutoff is applied; it is further supported by the reported trimerization features from the low-temperature refinement.

### Electrical transport in the modulated Te chain

The structural analysis above shows that the apparent infinite Te chain in the room-temperature *Pbcm* model is replaced at low temperature by a modulated and locally differentiated Te sublattice. This distinction has direct electronic consequences. If the uniform-chain average structure were electronically valid, the dispersive Te p bands crossing the Fermi level should produce metallic transport. Charge transport therefore provides a direct experimental test of whether LaCu_*x*_Te_2_ behaves according to the uniform-chain average model or according to the modulated local structure revealed by low-temperature crystallography. These measurements also allow us to distinguish the modulation in LaCu_*x*_Te_2_ from a conventional charge-density wave transition and to examine whether the modulated semiconducting state gives rise to unusual magnetotransport behavior at low temperatures.

**Fig. 5:**
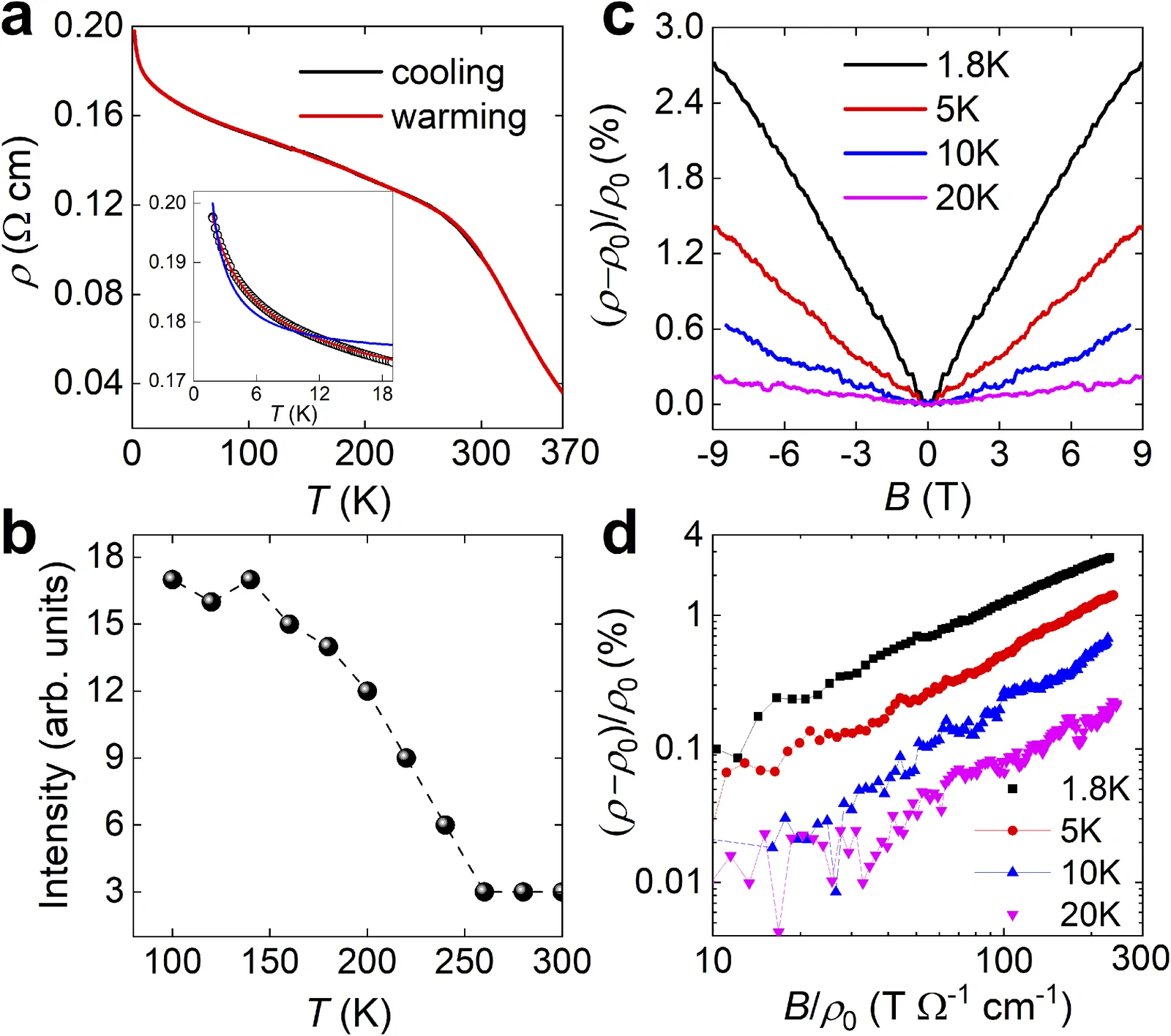
(a) Temperature dependence of resistivity from 1.8 to 370 K. The inset displays the *ρ vs. T* below 19 K. The blue and red solid lines in the inset present fits to the experimental data using Arrhenius-type conduction and three-dimensional VRH models, respectively. (b) Temperature-dependent intensity of the (0 4 0 −1) incommensurate diffraction peak. (c) Magnetoresistance behavior at low temperature. (d) Kohler's rule plots of the magnetoresistance of LaCu_*x*_Te_2_.

**Fig. 6:**
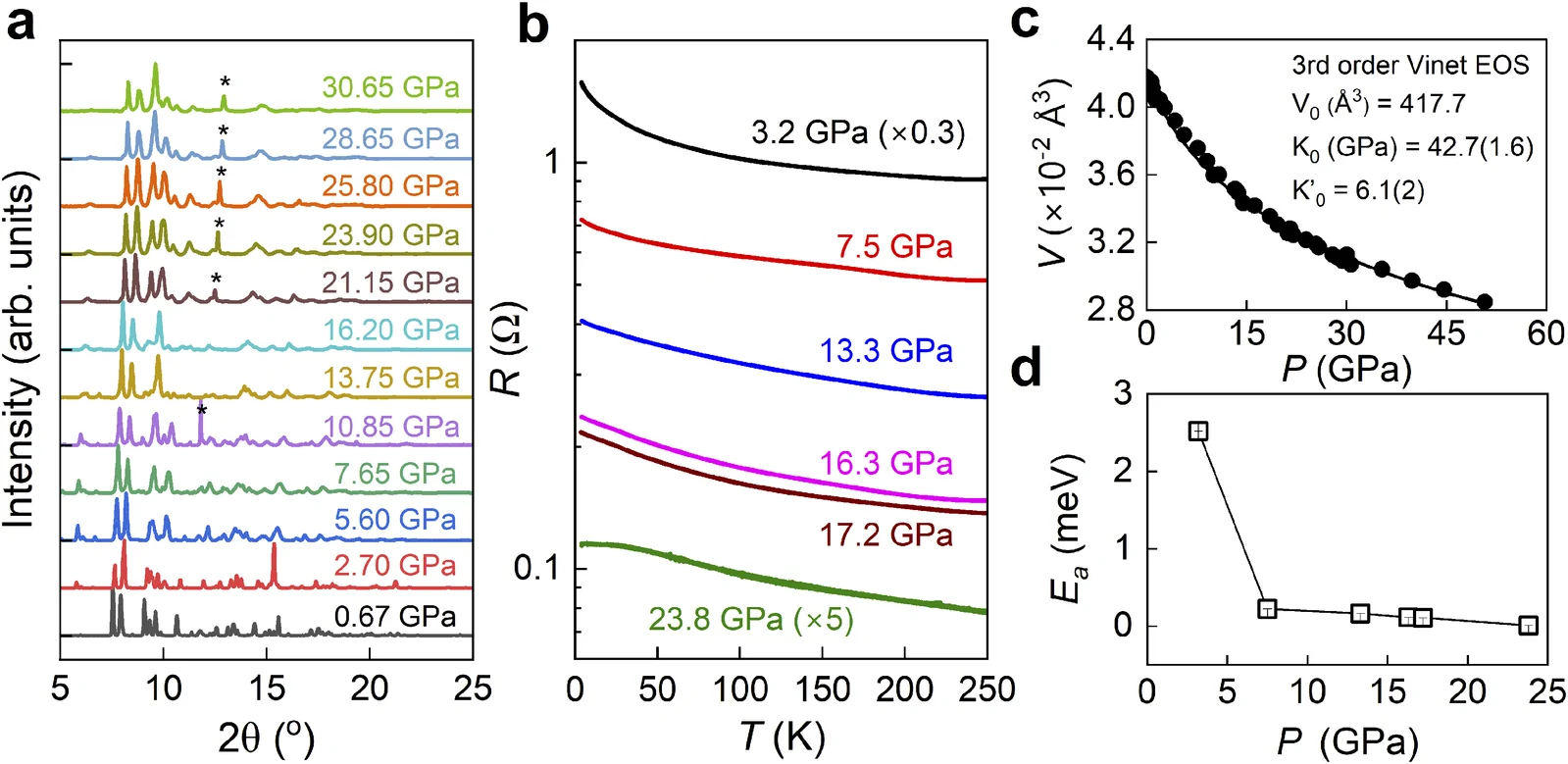
(a) Representative high-pressure diffraction patterns of LaCu_*x*_Te_2_ collected in a diamond-anvil cell. Marked additional peaks arise from solidified Ne. (b) Temperature-dependent resistance of single-crystalline LaCu_*x*_Te_2_ under selected pressures. The 3.2 GPa and 23.8 GPa curves have been multiplied by 0.3 and 5, for clarity. (c) Unit-cell volume as a function of pressure fitted using the Vinet equation of state. (d) Pressure dependence of the activation energy extracted from Arrhenius fits.

All transport properties in this study were measured on single-crystalline LaCu_*x*_Te_2_ samples. Fig. 5a shows the temperature dependence of electrical resistivity from 1.8 to 370 K. The resistivity increases upon cooling over the full measured temperature range, demonstrating semiconducting transport consistent with the previous report.^24^ However, unlike conventional band semiconductors, LaCu_*x*_Te_2_ shows only a modest change in resistivity, less than one order of magnitude across the measured temperature range. A second notable feature is a broad anomaly near 260 K, which is also observed in the high-pressure resistivity measurements discussed below (Fig. 6). To determine whether this feature is associated with the structural modulation, we tracked the temperature dependence of the incommensurate satellite peak (0 4 0 −1) in reciprocal space (Fig. S5). As shown in Fig. 5b, the satellite intensity decreases with increasing temperature, although residual intensity from the incommensurate modulation remains visible above 250 K. The correspondence between the broad resistivity anomaly and the temperature-dependent satellite intensity suggests that the structural modulation is coupled to charge transport.

In conventional charge–density-wave systems, the modulation is often an electronically driven instability associated with Fermi-surface nesting.^13,16,55^ Such transitions commonly produce a sharp resistive anomaly at the charge–density-wave transition temperature and may show hysteresis between cooling and warming cycles.^20,56^ In addition, the normal state above the charge–density-wave transition is typically metallic.^57–59^ LaCu_*x*_Te_2_ behaves differently. Its resistivity is semiconducting over the full measured temperature range, and the broad anomaly near 260 K appears without detectable hysteresis. These observations argue against a conventional electronically driven charge–density-wave transition and instead support a lattice-assisted, or phonon-coupled, origin of the incommensurate modulation.

To further clarify how the incommensurate modulation influences charge transport, we fitted the low-temperature region of the *ρ versus T* curve using Arrhenius-type, small-polaron, and variable-range hopping (VRH) models. The fitting results are shown in the inset of Fig. 5a and in Fig. S6. Among these models, three-dimensional VRH provides the best description of the experimental data from 1.8 to 19 K. VRH transport is commonly observed when electronic states near the Fermi level are localized by disorder, electronic interactions, or dimensional confinement.^60–62^ In LaCu_*x*_Te_2_, such localization is consistent with the combined effects of partial Cu occupancy and incommensurate modulation of the Te sublattice. As discussed above, the apparent chain is not a uniform infinite Te array, but is instead composed of discrete oligotelluride fragments whose relative populations are dictated by Cu deficiency and charge balance. This fragmentation disrupts continuous Te p-orbital overlap along the chain direction, localizing electronic states and leading to phonon-assisted hopping transport at low temperature. With increasing temperature, carriers can be thermally activated into more extended states, and the transport becomes better described by an Arrhenius-type process. Accordingly, the high-temperature region from 200 to 250 K was fitted using the Arrhenius model, yielding a small activation energy of 7.9 meV under ambient pressure. This small activation energy quantitatively captures the weakly activated nature of the semiconducting transport in the material.

Fig. 5c shows the magnetoresistance measured with the current parallel to the Te chain direction and the magnetic field perpendicular to the chains. Quasi-linear magnetoresistance is observed at the indicated temperatures and is reproducible across multiple samples (Fig. S7). Because LaCu_*x*_Te_2_ is a semiconductor with incommensurate structural modulation, this quasi-linear MR is unlikely to originate from quantum linear-magnetoresistance mechanisms typically associated with gapless or high-mobility electronic states.^63,64^ In addition, LaCu_*x*_Te_2_ exhibits nearly isotropic MR as the magnetic field is rotated from *H* perpendicular to the Te chains to *H* parallel to the Te chains (Fig. S7), making an anisotropic Fermi-surface origin unlikely.^65,66^ A more plausible framework is the mobility-disorder mechanism described by the Parish–Littlewood model.^67,68^ Unlike the original systems considered in this model, such as polycrystalline Ag_2+*δ*_Se, LaCu_*x*_Te_2_ is a single crystal with a well-defined average structure and an incommensurately modulated Te sublattice.

In this case, the incommensurate structural modulation and chemically differentiated Te network can generate spatial variations in carrier mobility, playing a role analogous to mobility disorder in polycrystalline systems. This provides a natural explanation for the quasi-linear MR observed in single-crystalline LaCu_*x*_Te_2_ and suggests that Parish–Littlewood-type behavior can arise from intrinsic crystallographic modulation, without requiring extrinsic grain or compositional disorder. Kohler's law provides a scaling test for whether the magnetotransport is governed by a single dominant scattering time.^69,70^ As shown in Fig. 5d, the MR curves do not collapse onto a single Kohler scaling curve, indicating that the transport cannot be described by a single scattering time. This behavior is consistent with spatially heterogeneous mobility arising from the modulated and chemically differentiated Te sublattice.

### High-pressure structure and transport of the modulated Te chain state

High pressure also provides a useful test of the robustness of the incommensurately modulated state and helps distinguish LaCu_*x*_Te_2_ from conventional charge–density-wave systems, where pressure often suppresses the ordered state and can restore metallicity. We thus carried out high-pressure characterizations to examine whether pressure can close the semiconducting gap by enhancing Te–Te orbital overlap and modifying the modulated Te sublattice. All diffraction patterns collected below 30.65 GPa are shown in Fig. S8. As shown in Fig. 6a, the high-pressure diffraction patterns are well described by the *Pbcm* average structure over the measured pressure range. The additional marked peaks arise from solidified neon used as the pressure-transmitting medium. No new Bragg reflections or peak splittings indicative of a pressure-induced structural phase transition are observed, demonstrating the high structural stability of LaCu_*x*_Te_2_ under compression. Unit-cell volumes extracted from the refinements are plotted as a function of pressure in Fig. 6c. The pressure–volume data were fitted using the Vinet equation of state:^71,72^2
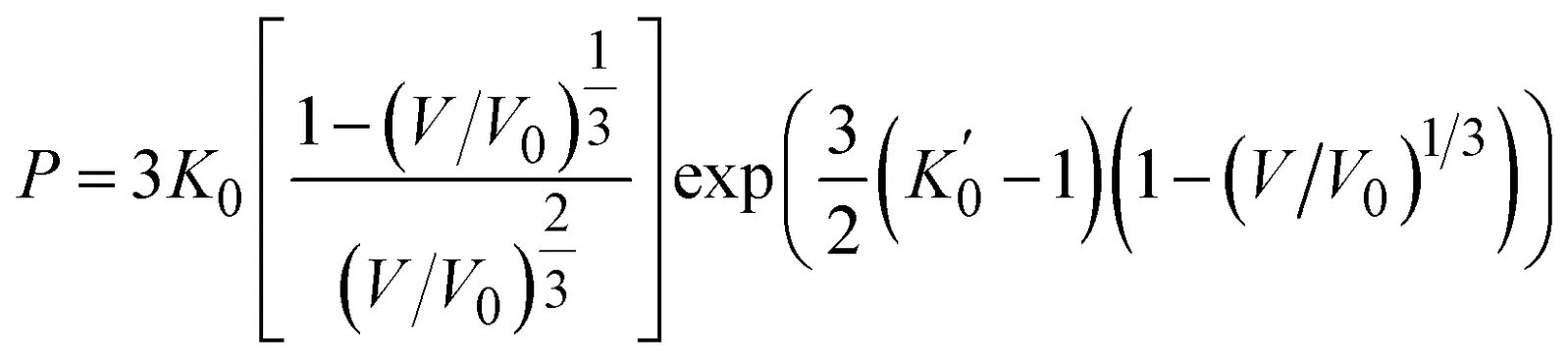
where *K*_0_, 
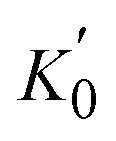
, *V*_0_ represent the zero-pressure bulk modulus, its pressure derivative, and the zero-pressure unit-cell volume, respectively. As shown in Fig. 6c, the experimental data conform well with the fitting curve with parameters *K*_0_ = 42.7 GPa, 
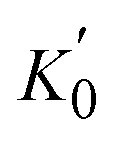
 = 6.1, and *V*_0_ = 417.7 Å^3^, showing continuous compression without a phase transition over the measured range up to 30.65 GPa. Despite of the absence of van der Waals characters in LaCu_*x*_Te_2_, the ratio *V*(50 GPa)/*V*_0_ ∼69.0% suggests a high compressibility which is comparable to low-dimensional materials.

Fig. 6b shows the temperature dependence of resistance under pressure. The resistance upturn is progressively suppressed with increasing pressure, indicating a pressure-induced decrease in the transport activation energy. Arrhenius fits to the 50–150 K region yield activation energies that decrease systematically with pressure (Fig. 6d), consistent with pressure-enhanced orbital overlap and partial closing of the transport gap. Nevertheless, semiconducting behavior persists up to the highest measured pressure of 23.8 GPa. At lower temperatures, from base temperature to 20 K, the resistance remains best described by a three-dimensional VRH model among the semiconducting transport models considered, indicating that localized transport survives even under substantial compression.

The persistence of semiconducting transport under pressure is significant because it shows that the semiconducting state is not readily driven into the metallic state predicted for the idealized uniform-chain structure. Instead, the modulated and chemically differentiated Te sublattice remains electronically consequential under compression, continuing to interrupt the continuous Te p-orbital pathway needed for metallic transport. This behavior further distinguishes LaCu_*x*_Te_2_ from conventional charge–density-wave materials, in which pressure often suppresses the electronically driven instability and restores a metallic state. In LaCu_*x*_Te_2_, the pressure response instead supports a robust lattice-coupled modulation associated with Te chain fragmentation and electronic localization. Chemical doping, rather than pressure alone, may therefore be a more effective route for introducing carriers and exploring possible emergent metallic, charge-density wave or superconducting states in this system.

## Conclusions

Instead of conventional infinite linear tellurium chains, LaCu_*x*_Te_2_ presents intrinsic tellurium speciation and disorder within an average one-dimensional motif. Low-temperature single-crystal diffraction uncovers an incommensurate modulation, while total X-ray scattering analysis provides evidence for local unit-cell tripling along the direction of the apparent Te chain. When these results are considered together with charge balance, the average chain is more plausibly described as a statistically disordered collinear arrangement of monotelluride and oligotelluride species, most notably linear [Te_3_]^4−^ fragments embedded within a matrix of Te^2−^ centers. The substantial Cu deficiency requires partial oxidation of the tellurium sublattice, consistent with local mixtures of monotelluride and oligotelluride units. Thus, the crystallographic chain is not a true infinite covalent Te chain, but an average structural manifestation of locally distinct tellurium anions distributed along one crystallographic direction.

This reinterpretation resolves the long-standing inconsistency between calculations based on an idealized undistorted chain and the experimentally observed semiconducting behavior. The transport data, including variable-range hopping at low temperature and nonsaturating linear magnetoresistance, are consistent with carrier localization arising from structural and occupational disorder and not from a canonical electronically driven charge–density-wave ground state. High pressure reduces the transport activation energy but does not induce a structural transition at room temperature, showing that the semiconducting state is robust even under substantial compression. These conclusions may extend to other LnCu_*x*_Te_2_ compounds, suggesting that they may also contain short linear polytellurides as chemically distinct local anionic species. LaCu_*x*_Te_2_ therefore provides an instructive example showing that incommensurate modulation in low-dimensional chalcogenides need not signify a charge–density-wave instability, but may instead reflect a chemically driven, locally disordered route to structural and electronic stabilization.

## Author contributions

M. G. K. and D. Y. C. conceptualised this work, designed the experiments, and supervised the project. Y. W., T. S. I., and Shima Shahabfar drafted the initial manuscript. Y. W. carried out the room-temperature SXRD, ambient-pressure electrical transport measurements, and analysed the data. T. S. I., A. B., and C. D. M. carried out the variable-temperature SXRD and analysed the data. C. H., F. S., J. S. S., N. P. S., and R. J. H. carried out the high-pressure XRD and analysed the data. Shima Shahabfar and C. W. carried out the DFT calculations. John Bakir, A. G., and S. D. carried out the high-pressure electrical transport measurements. H. Z., Joohee Bang, and S. R. carried out the X-ray total scattering characterizations and analysed the data. J. M. H., P. A., D. S., H. Z., and Y. W. carried out the powder PDF and analysed the data. The crystals in this study were synthesized by Y. W., H. Z., and Souvik Sasmal. All authors proofread and contributed to the final version of the manuscript.

## Conflicts of interest

The authors declare no competing financial interests.

## Supplementary Material

SC-OLF-D6SC05234G-s001

SC-OLF-D6SC05234G-s002

## Data Availability

CCDC 2559818 and 2561601 contain the supplementary crystallographic data for this paper.^73*a*,*b*^ The room-temperature average structure was previously reported in ref. 24. The data supporting this article have been included as part of the supplementary information (SI). Supplementary information: additional calculated phonon dispersion of LaCu_0.5_Te_2_; Rietveld refinements of the LaCu_0.5_Te_2_ powder X-ray diffraction patterns; experimental and deconvoluted powder PDF patterns; total X-ray scattering reflection patterns at 265 K; X-ray diffraction patterns along the (010) reciprocal-space section at different temperatures; temperature-dependent resistivity data analyzed using different models; linear MR behaviors at 1.8 K; high-pressure X-ray diffraction data up to 30.7 GPa; DFT-calculated formation energies of phases in the La–Cu–Te system; and detailed crystallographic information for the room-temperature and 100 K structures. See DOI: https://doi.org/10.1039/d6sc05234g.
