# Supplementary material for: Hidden tellurium speciation resolves the paradox of infinite Te chains in LaCuxTe2
Source: Chem Sci. 2026 Aug 14;17(39):18987–98. doi: 10.1039/d6sc05234g (PMC13507780; doi:10.1039/d6sc05234g)
Supplement: Supplementary file 1 [file SC-017-D6SC05234G-s001.pdf]

Supplementary Information (SI) for Chemical Science.
This journal is © The Royal Society of Chemistry 2026

Electronic supplementary information

Hidden Tellurium Speciation Resolves the Paradox of Infinite Te Chains in $LaCu_xTe_2$

Yihao Wang,[ab] Thomas S. Ie,[b] Clayton Halbert,[c] Shima Shahabfar,[d] John Bakir,[e] Hengdi Zhao,[a] Joohee Bang,[a] Justin M. Hoffman,[f] Fatemeh Safari,[g] Paribesh Acharyya,[b] Debattam Sarkar,[b] Adam Balvanz,[b] Anukriti Ghimire,[e] Souvik Sasmal,[a] Christos D. Malliakas,[b] Jesse S. Smith,[f] Nilesh P. Salke,[g] Stephan Rosenkranz,[a] Shanti Deemyad,[e] Christopher Wolverton,[d] Russell J. Hemley,[acgh] Duck Young Chung,[a] and Mercouri G. Kanatzidis[ab*]

[a] *Materials Science Division, Argonne National Laboratory, Lemont, Illinois 60439, United States*
[b] *Department of Chemistry, Northwestern University, Evanston, Illinois 60208, United States*
[c] *Department of Chemistry, University of Illinois Chicago, Chicago, Illinois 60607, United States*
[d] *Department of Materials Science and Engineering, Northwestern University, Evanston, Illinois 60208, United States*
[e] *Department of Physics and Astronomy, University of Utah, Salt Lake City, Utah 84112, United States*
[f] *X-ray Science Division, Argonne National Laboratory, Lemont, Illinois 60439, United States*
[g] *Department of Physics, University of Illinois Chicago, Chicago, Illinois 60607, United States*
[h] *Department of Earth and Environmental Sciences, University of Illinois Chicago, Chicago, Illinois 60607, United States*

* Corresponding Author
E-mail: m-kanatzidis@northwestern.edu

EXPERIMENTAL METHODS

Synthesis. $LaCu_xTe_2$ polycrystals were synthesized by a two-step direct combination method. La (0.2074g, 1.4931 mmol) and Te (0.3926g, 3.0768 mmol) powders at a molar ratio of 1:2.06 were weighed and thoroughly mixed in an agate mortar. Here the slight excess of Te was adopted to compensate for its evaporation at high temperatures. Then, the mixture was flame sealed in an evacuated fused silica tube and annealed using a programmable resistive box furnace. The mixture was heated to 200 °C held for 2 hours, then heated to 350 °C held for 6 hours, then heated to 450 °C held for 12 hours, and finally heated to 800 °C held for 4 days. PXRD analysis confirmed the high purity of $LaTe_2$ (Figure S2(a)). The authors note that avoiding La oxidation is crucial to ensure purity of the product, because oxygen is unremovable once introduced to the system and will lead to failure of the subsequent reaction.

Before synthesizing $LaCu_xTe_2$ polycrystals, copper powder was activated by soaking in diluted HCl (0.4 mol/L) for 5 minutes to remove the CuO on the surface of particles. Then the Cu powder was rinsed with methanol to remove HCl, subsequently dried under vacuum. $LaTe_2$ (0.2313g, 0.5870 mmol) and Cu (0.0187g, 0.2944 mmol) powders at a molar ratio of 1:0.5 were thoroughly mixed and pressed into pellet under a pressure of 150 MPa. The pellet was sealed in an evacuated fused silica tube and annealed at 450 °C for 12 hours, then heated to 600 °C and held for 3 days before cooling to room temperature. Rietveld refinements (Fig. S2(c)-(d)) on the PXRD pattern collected from the resulting powder demonstrate that the sample is composed of phase-pure $LaCu_xTe_2$ polycrystals. It should be noted that using $LaTe_2$ as a precursor and pressing the starting materials into pellet are critical in acquiring pure $LaCu_xTe_2$ polycrystals. Reactions with loose powder of La, Cu, and Te as starting materials yield a mixture of $LaCu_{0.5}Te_2$ and $LaTe_2$ as major phases and ternary $La_3Cu_5Te_7$ as a minor phase (Figure S2(b)).

Single-crystalline $LaCu_xTe_2$ samples were synthesized by using a KI-flux method. La (0.1389g, 1mmol), Cu (0.0318g, 0.5mmol), and Te (0.2552g, 2mmol) powders at a stoichiometric molar ratio of La:Cu:Te = 1:0.5:2 were weighed and thoroughly mixed with KI (0.4g, 2.4mmol) in an agate mortar. Then the mixture was loaded in an alumina crucible and sealed in a niobium tube and then a fused silica tube. The tube was heated to 820 °C, held for 96 hours, and cooled down to 600 °C at a rate of 0.3 °C /h. The excess KI flux was removed by soaking the product in distilled water for ten minutes.

The nominal starting composition was La:Cu:Te = 1:0.5:2; however, refinements of individual single crystals consistently indicate Cu-deficient compositions. The exact Cu content varies somewhat among crystals and between room-temperature and low-temperature refinements, consistent with a finite Cu compositional width in $LaCu_xTe_2$. This variation is chemically significant because x directly controls the average oxidation state of the tellurium sublattice and therefore the relative population of isolated telluride and oligotelluride motifs.

Structural Characterization and Refinements. Finely ground powder was mounted on a flat Si substrate, then measured on a Rigaku MiniFlex diffractometer with a Cu Kα radiation source (λ = 1.5406 Å) to collect the powder X-ray diffraction (PXRD) data. After data collection, Rietveld refinements were carried out by using the GSAS-II package[1] to identify the products and analyze their purity. The observed pattern from PXRD was compared with the pattern generated based on the $LaCu_xTe_2$ cif file from ICSD database. The refinement results were exhibited in Fig. S1.

Room-temperature single crystal X-ray diffraction (SXRD) data were collected on a STOE IPDS 2T diffractometer with Mo Kα radiation (λ = 0.71071 Å), operated at 50 kV and 40 mA. Data reduction was performed using the X-Area software package, applying a numerical absorption correction via X-Shape. The structure was solved using ShelXT[2] with Intrinsic Phasing solution method, and refined by using full-matrix least-squares method using ShelXL.[3] Solution and refinement were operated within the Olex2 graphical interface.[4]

Low-temperature SXRD was collected on an XtaLAB Synergy diffractometer equipped with a micro-focus sealed X-ray tube PhotonJet (λ = 0.71071 Å) and a Hybrid, running at 65 kV and 0.68 mA, and a Dectris Pilatus3 R CdTe 300K Hybrid Photon Counting detector. The temperature of the crystal was controlled with an Oxford Cryosystems cryostat. Data reduction was performed with the CrysAlisPro software using an empirical absorption correction. As the material was incommensurately modulated, the reflection file was imported into Jana2006[5] where a solution was achieved using SUPERFLIP[6] and further refined using a least squares approach, on F^2, as implemented in Jana2006. There was no evidence of any occupation modulation on any of the La or Te atoms and thus were exclusively refined using a single positional wave. On the other hand, Cu was refined using both positional and occupational waves. Modulation of the thermal displacement parameters was modeled using waves on an atom-to-atom basis. Additionally, as the sub cell saw a reduction in symmetry, a twin domain (twin law [1 0 0, 0 -1 0, 0 0 -1]) was additionally refined.

Variable temperature SXRD data were collected on a STOE StadiVari diffractometer equipped with an AXO Ag Kα micro-focus sealed X-ray A-MiXS source (λ = 0.560834 Å), running at 65 kV and 0.68 mA, and a Dectris Pilatus3 R CdTe 300K Hybrid Photon Counting detector. Temperatures were maintained through the usage of an Oxford Cryostream 700 plus low temperature device.

Pair Distribution Function Analysis Total scattering data was collected at Beamline 11-IB-D at the Advanced Photon Source (APS) at Argonne National Laboratory with a photon energy of 58.6 keV (0.2116 Å). The calibration of the measurement is carried out using NIST-traceable SRM 674b CeO_2 material loaded into a Kapton capillary mounted in the same distance from the detector as the samples. Data has been collected on a PerkinElmer 1621 a-Si detector using 1200 0.1- second exposures summed into one image for a total collection time of 2 minutes. The length of the exposure has been optimized to yield satisfactory signal quality without overexposing the detector. Data has been acquired using the QXRD program; dark current and flat-field corrections have been applied within the same software.

The two-dimensional total-scattering images were reduced in GSAS-II using the CeO_2 standard collected at the same sample-to-detector distance to calibrate the detector geometry, followed by masking of capillary/streak artifacts and azimuthal integration to obtain the corresponding one-dimensional total-scattering patterns. The reduced scattering data were Fourier transformed to generate the pair distribution function, G(r), using Q_{max} = 15.4894 Å^{-1}. Real-space refinements were then carried out in PDFgui/PDFfit2 programs[7] using the $LaCu_{0.4}Te_2$ structural model, with the Cu occupancies fixed at 0.20 on the expanded Cu sites to preserve the overall composition. Refinements were evaluated over multiple real-space windows (1.8−20, 1.8−30, and 1.8−60 Å); the shortest-range refinement over 1.99−20.01 Å gave R_w = 0.15, whereas the best overall fit was obtained over the intermediate range, which was therefore used for the final analysis. GSAS-II software[1] was used for detector calibration, image integration, and PDF generation, while PDFgui/PDFfit2 was used for real-space modeling and refinement.

Total-scattering Experiments Total scattering data were collected at Sector 6-ID-D of the APS using a monochromatic X-ray energy of 90.1 keV and a Pilatus CdTe 2M pixel area detector. The sample was rotated 360° about its surface normal with the rotation axis perpendicular to the incident beam, in 0.1° steps with an exposure time of 1 s per frame at a sample-to-detector

distance of 702 mm. To fill inter-module detector gaps and maximize reciprocal-space coverage, the step-scan was repeated at three ω offset angles (−15°, 0°, and 15°).

High-Pressure Structure High-pressure experiments were carried out using symmetric diamond anvil cells (DACs) with 300 µm culets. Crystal grains of $LaCu_xTe_2$ were placed in a Rhenium gasket preindented to 35 µm thickness. Ruby and gold were added to serve as pressure calibrants before neon gas was loaded as the pressure-transmitting medium. High-pressure angle dispersive X-ray diffraction measurements were carried out at beamline IDB, sector 16, HPCAT of the Advanced Photon Source (APS) at Argonne National Laboratory (ANL) using a wavelength of 0.42459 Å. Diffraction data was analyzed using Dioptas[8] and lattice parameters were obtained by Le Bail refinement[9] using the Jana2020[5] software packages. Pressure-volume data were fitted to Vinet equations of state[10] in the software EosFit-7c[11] and EosFit GUI[12].

Electrical Transport Measurements Electrical transport measurements at ambient pressure were carried out on a Physical Properties Measurements System (Dynacool 9T, Quantum Design). To avoid the large contacting resistance caused by the sensitivity between silver paste and tellurides, we deposited gold on selected surface of $LaCu_xTe_2$ single crystals using homemade photomasks[13] and a sputter coater (Leica EM ACE600). Then gold wires with a diameter of 25 µm were attached to the coated areas using Dupont 4929N silver paint. This intermediate layer between the sample and silver paint decreased the contacting resistance down to tens of Ohms. To eliminate the influence caused by misalignments of electrodes, the magnetoresistance was measured at both negative and positive magnetic fields.

High-Pressure Electrical Transport Electrical transport measurements of single-crystal $LaCu_xTe_2$ under pressures were conducted using the four-probe method in a van der Pauw configuration, with NaCl serving as the pressure-transmitting medium. All measurements were carried out in a diamond anvil cell (DAC) with 350 µm culet anvils. Stainless steel gaskets were pre-indented to a thickness of ~65 µm, and a ~150 µm diameter hole was drilled and filled with an alumina–epoxy mixture as an insulating material, then repressed to ~65 µm thickness. A ~120 µm hole was subsequently laser-drilled in the compressed insulated gasket.

The pressure chamber was filled with fine NaCl powder as the pressure-transmitting medium and compressed until it became transparent. Owing to its relatively low bulk modulus (23.8 GPa), cubic crystal structure, and nearly isotropic compressibility, NaCl provides a quasi-hydrostatic environment for these measurements. A piece of single-crystal $LaCu_xTe_2$ was then carefully

loaded on top of the transparent NaCl layer, and four platinum electrodes were arranged to contact the sample.

Temperature-dependent resistance measurements were performed in a closed-cycle cryocooler system over the temperature range 3.4–300 K, using a Keithley 6221 DC current source supplying 200 μA and a Keithley 2182A nanovoltmeter. Pressure was determined using the ruby fluorescence method at both room temperature and low temperature (~4 K) with the aid of an online ruby fluorescence system.

Computational Methods Density functional theory (DFT) calculations were performed using the Vienna Ab initio Simulation Package (VASP)[14] within the projector augmented wave (PAW) formalism. The exchange-correlation potential was described by the Perdew–Burke–Ernzerhof (PBE) functional.[15] To account for the partial Cu occupancy in the triclinic lattice, we enumerated all symmetrically distinct sites for a single copper atom within the unit cell; the lowest energy configuration was adopted as the structural model. We used the Open Quantum Materials database (OQMD) to assess the thermodynamic stability of $LaCu_{0.25}Te_2$.[16]

A plane-wave energy cutoff of 600 eV and ∼3000 *k*-points per reciprocal lattice were used for structural relaxation and static calculations. Convergence criteria were set to 10^{-7} meV for the total energy and 0.01 meV/Å for ionic forces during relaxation. Spin-orbit coupling (SOC) was included in the calculation of the electronic band structure and density of states (DOS). Phonon calculations were carried out using the finite displacement method as implemented in the Phonopy code, utilizing a 104-atom supercell and a 2 × 2 × 2 *k*-point grid.[17]

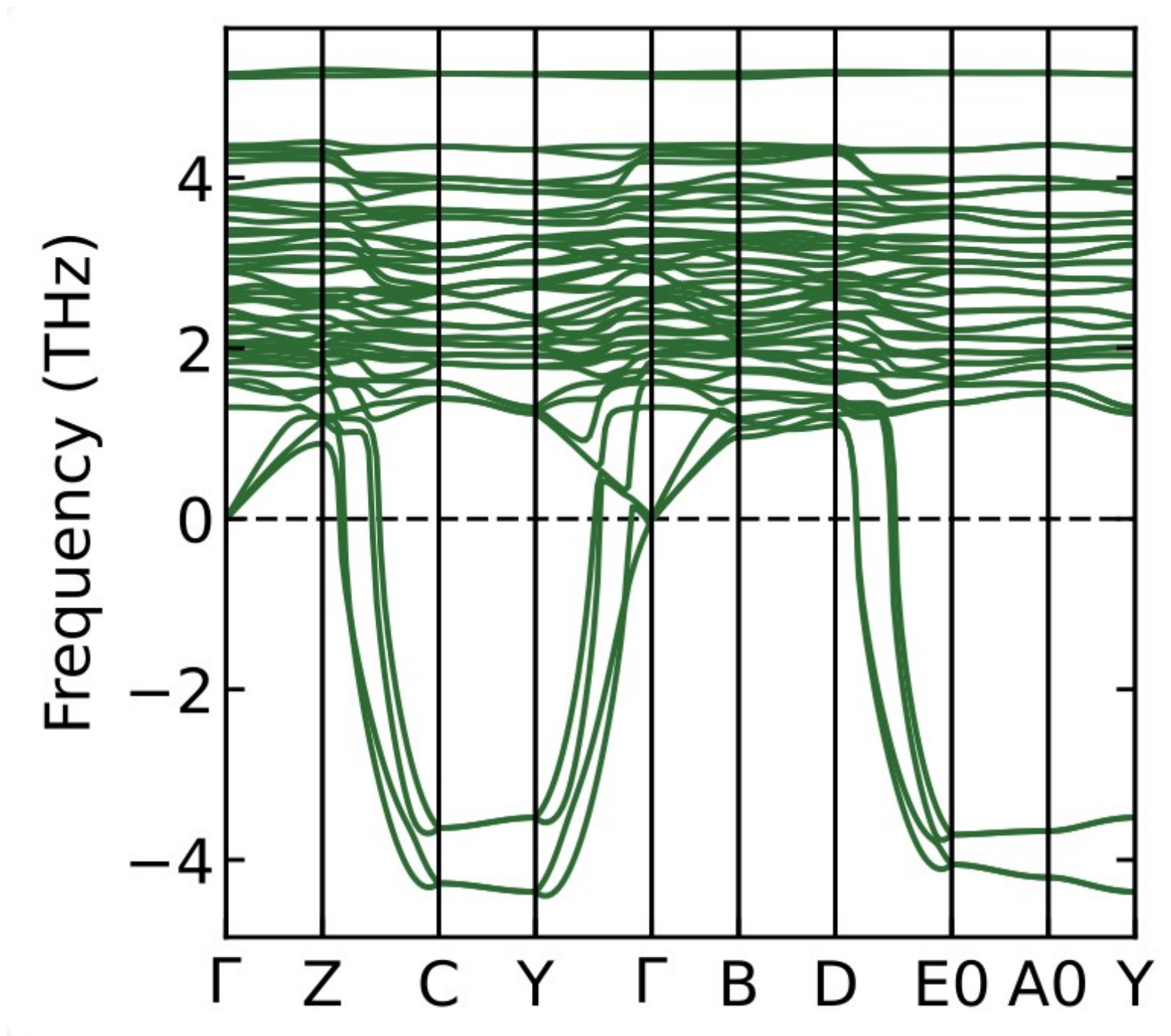

Fig. S1. Calculated phonon dispersion of La_2CuTe_4, corresponding to $LaCu_{0.5}Te_2$, showing imaginary modes that indicate a tendency toward structural instability.

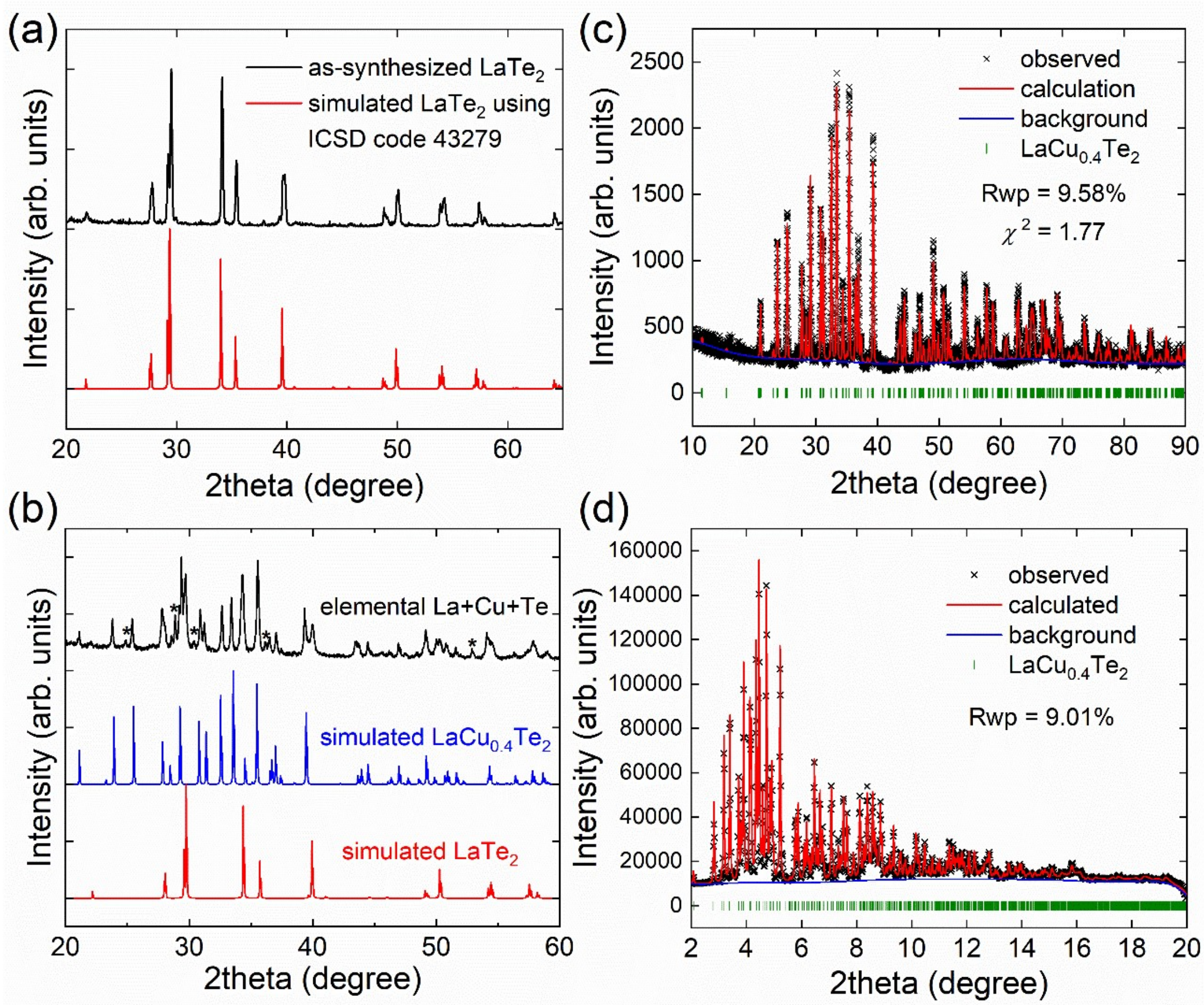

Fig. S2. (a) PXRD pattern of $LaTe_2$ synthesized by the procedure described in the experimental method section. (b) PXRD patterns of the product from annealing stoichiometric La, Cu, and Te powder (top), the simulated $LaCu_{0.4}Te_2$ (middle), and the simulated $LaTe_2$ (bottom). The additional asterisk-marked diffraction peaks indicate the existence of $La_3Cu_5Te_9$ as a minor phase in the product. (c) Room-temperature PXRD pattern of $LaCu_xTe_2$ collected on Rigaku MiniFlex diffractometer and corresponding Rietveld refinements using GSAS-II. (d) Room-temperature PXRD pattern transformed from total scattering data collected at Beamline 11-IB-D at the Advanced Photon Source and corresponding Rietveld refinements using GSAS-II. The good agreement between the experimental diffraction patterns and refinement is indicative of high purity of $LaCu_xTe_2$ powder.

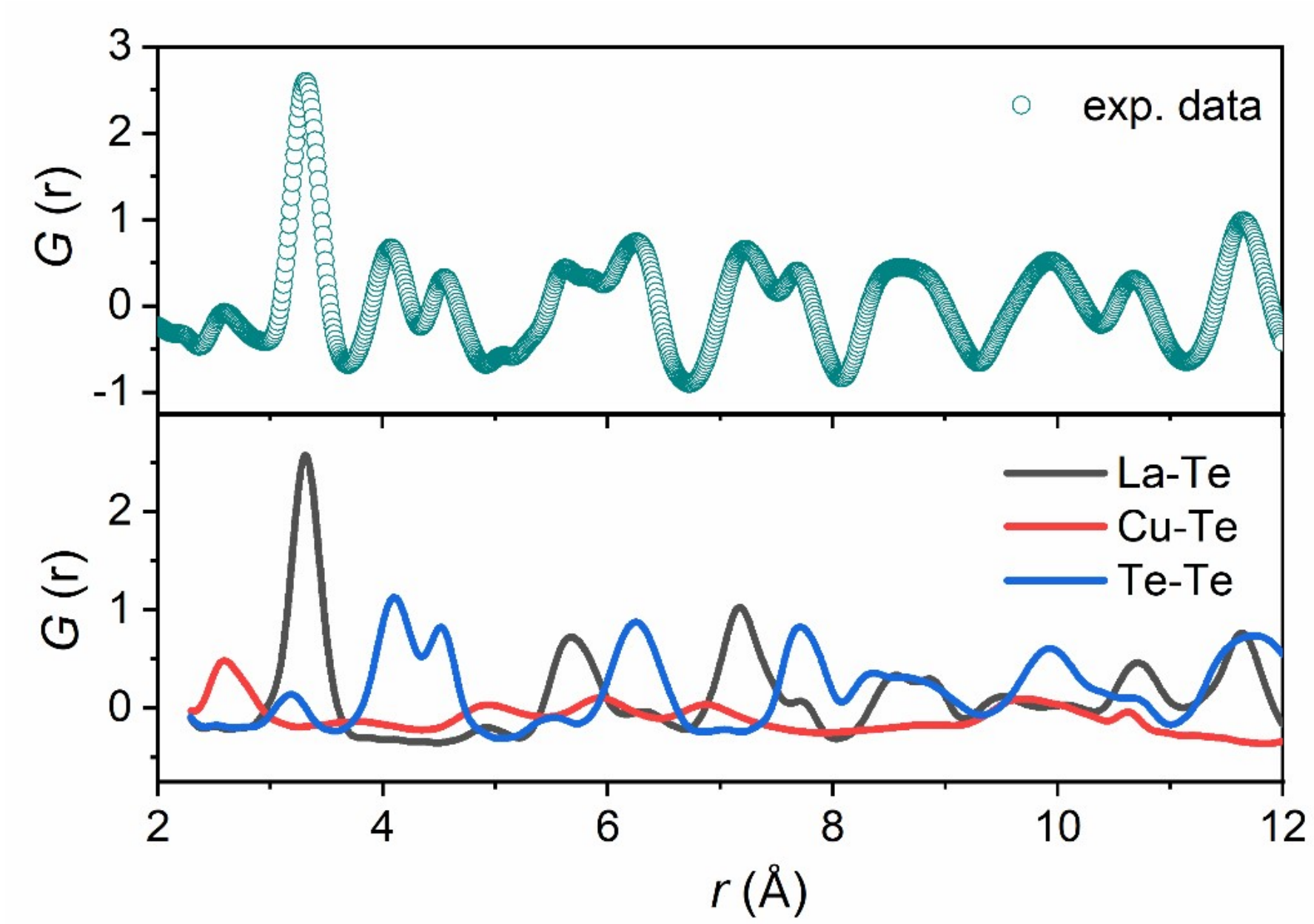

Fig. S3. Upper plot: Experimental PDF pattern up to 12 Å of the $LaCu_{0.5}Te_2$ powder. The dataset was collected at room temperature. Lower plot: Deconvoluted PDF for each bonding contribution.

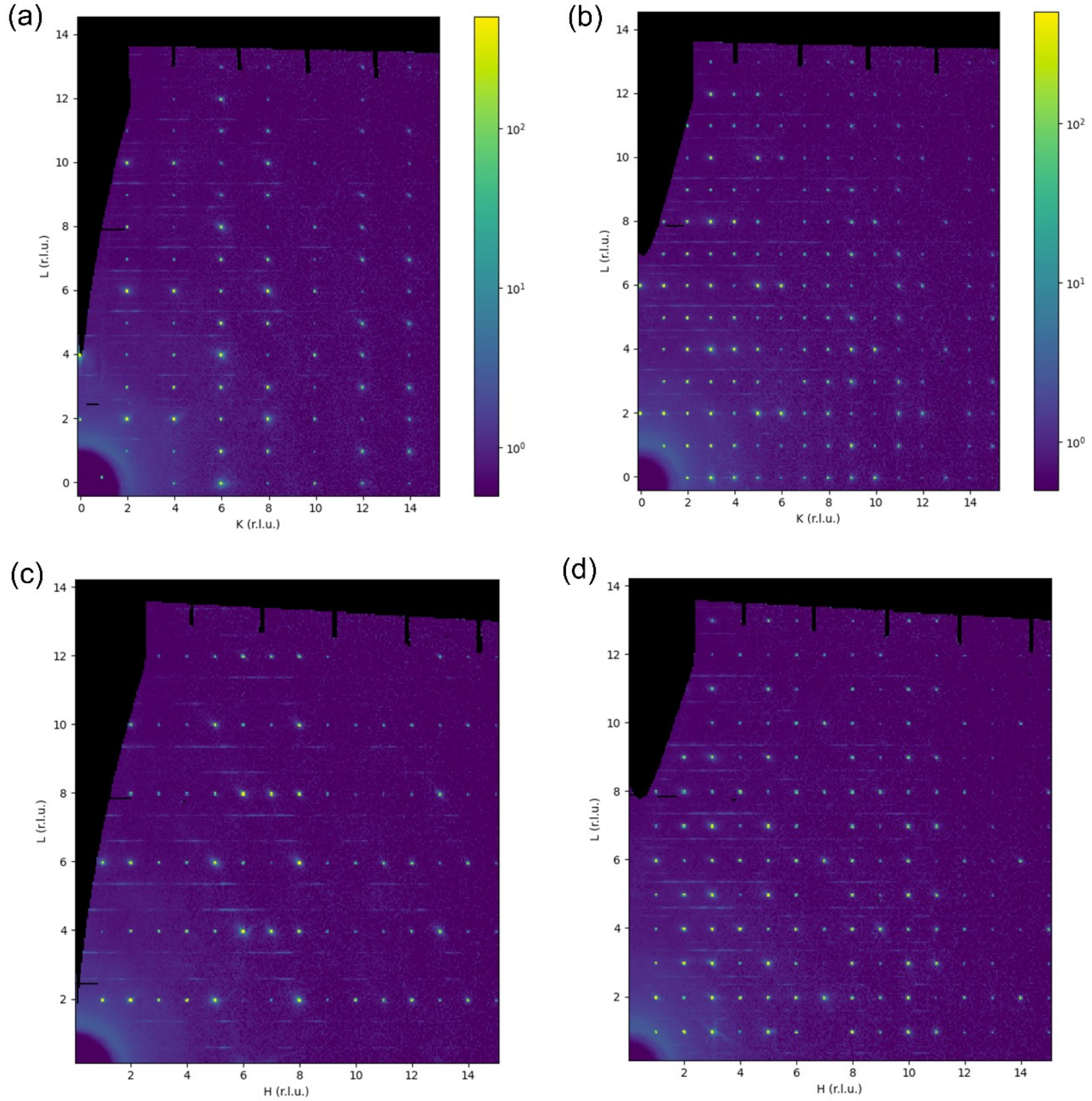

Fig. S4. Total X-ray scattering reflection patterns at 265 K along the (a) 0*kl* plane; (b) 1*kl* plane; (c) *h*0*l* plane; (d) *h*1*l* plane. Diffuse scattering streaks appear periodically along *l*, i.e., Te-chains direction, with a spacing of 1/3.

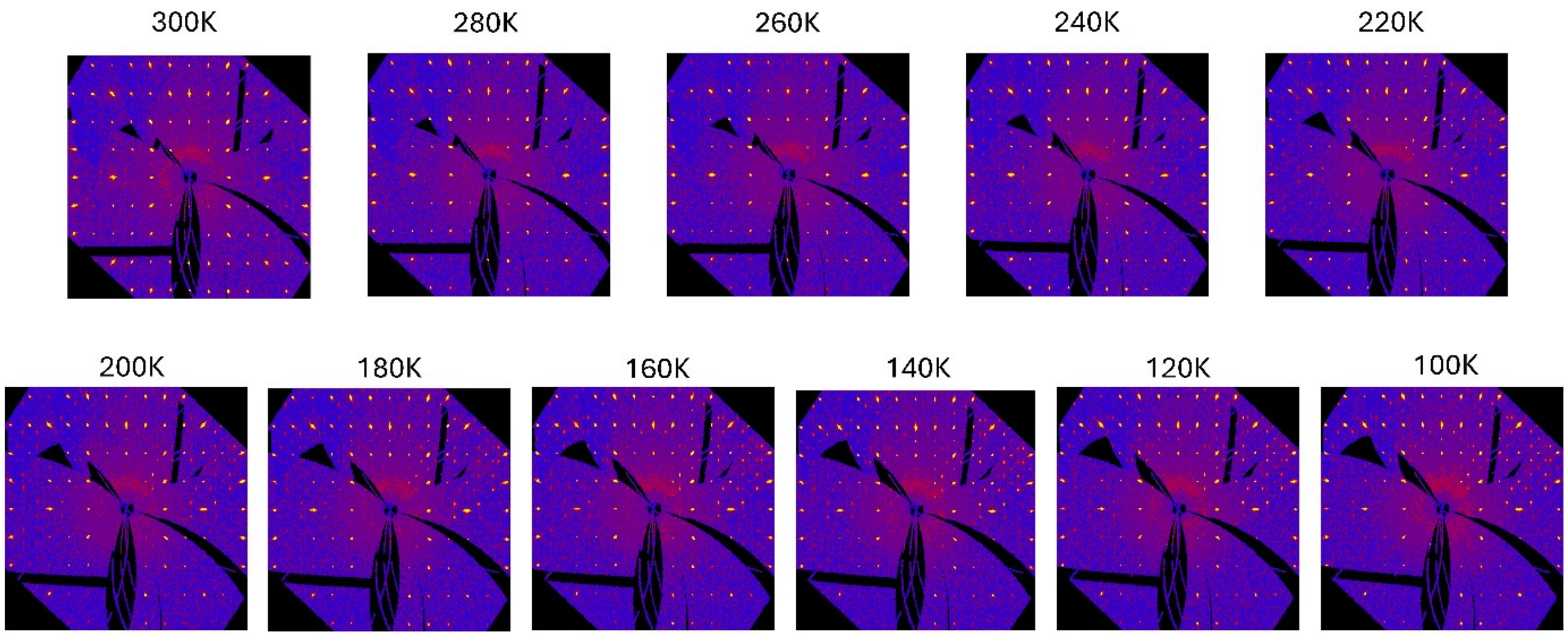

Fig. S5. The diffraction pattern along the (010) reciprocal-space section at different temperatures. The intensity of diffraction peaks from average structure remains stable, while the intensity of diffraction peaks from incommensurate modulated structures decreases as increasing temperatures.

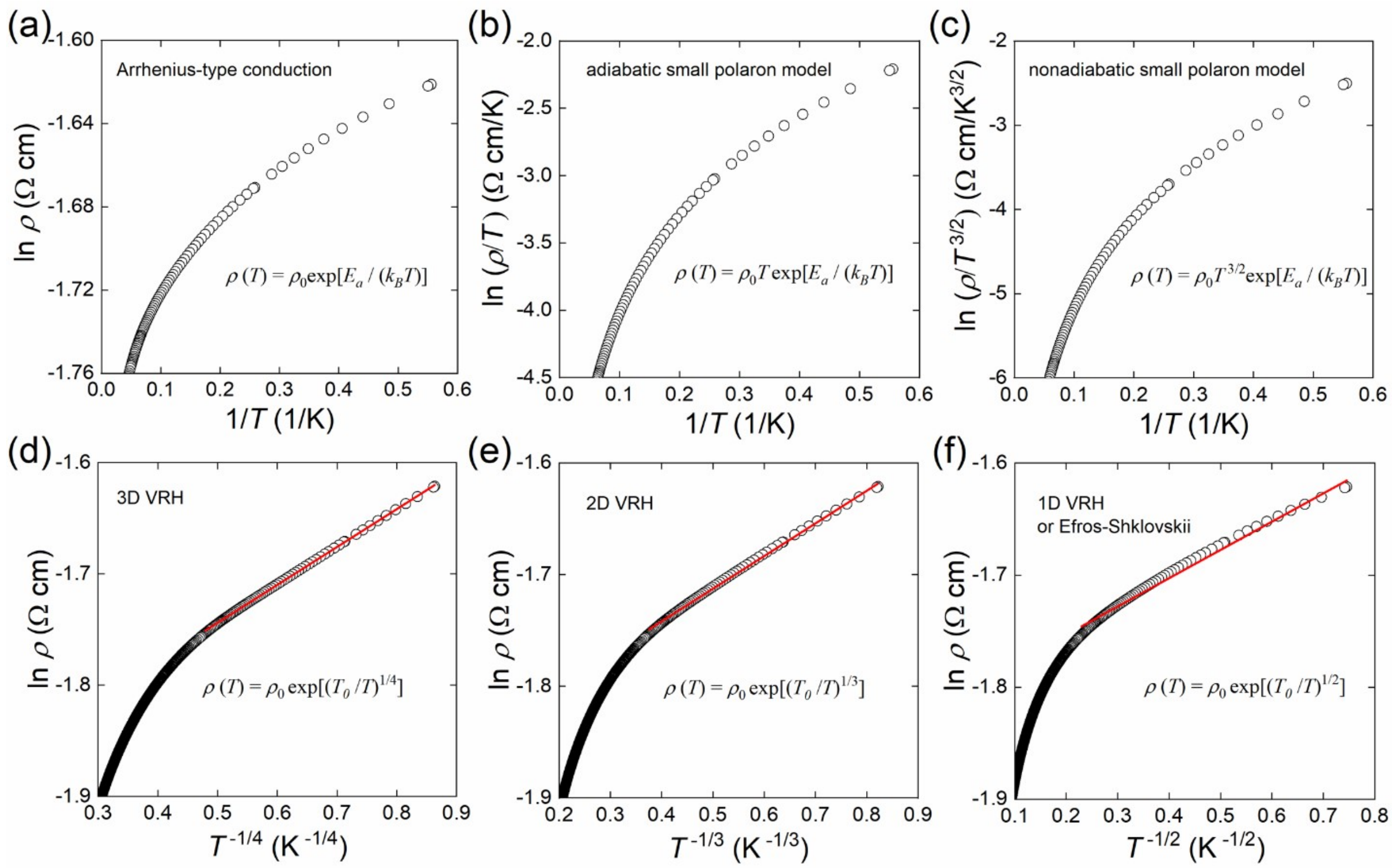

Fig. S6. The resistivity behavior plotted in forms following indicated different models. (a) Arrhenius-type conduction model; (b) adiabatic small polaron model; (c) nonadiabatic small polaron model. No linearity shows up in the temperature region from 1.8 K to 20 K in Fig. S6(a)-(c), indicating that the dominant mechanism in $LaCu_xTe_2$ does not conform with the models. (d) 3D VRH model; (e) 2D VRH model; (f) 1D VRH or Efros-Shklovskii VRH model. The red solid lines in Fig. S6(d)-(f) indicate linear fits from 1.8 K to 19 K, which explicitly demonstrates that the 3D VRH model gives the best description to the experimental data.

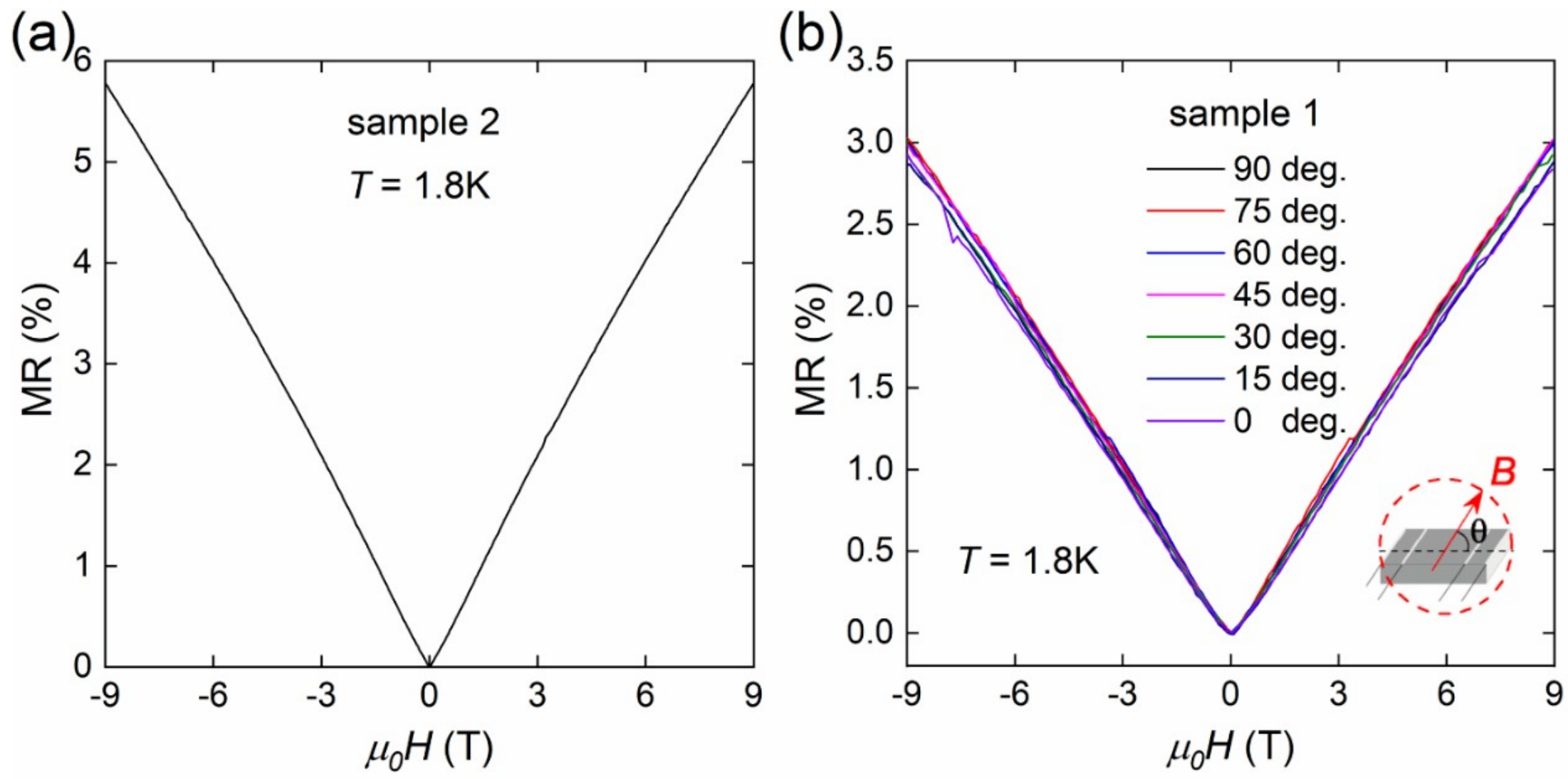

Fig. S7. (a) Magnetotransport behavior at 1.8 K measured on a second $LaCu_xTe_2$ single crystal (sample 2) with magnetic fields up to 9 T. (b) Magnetoresistance at 1.8 K with magnetic field along different directions. As shown in the inset, θ denotes the angle between applied magnetic field and in-plane electrical current.

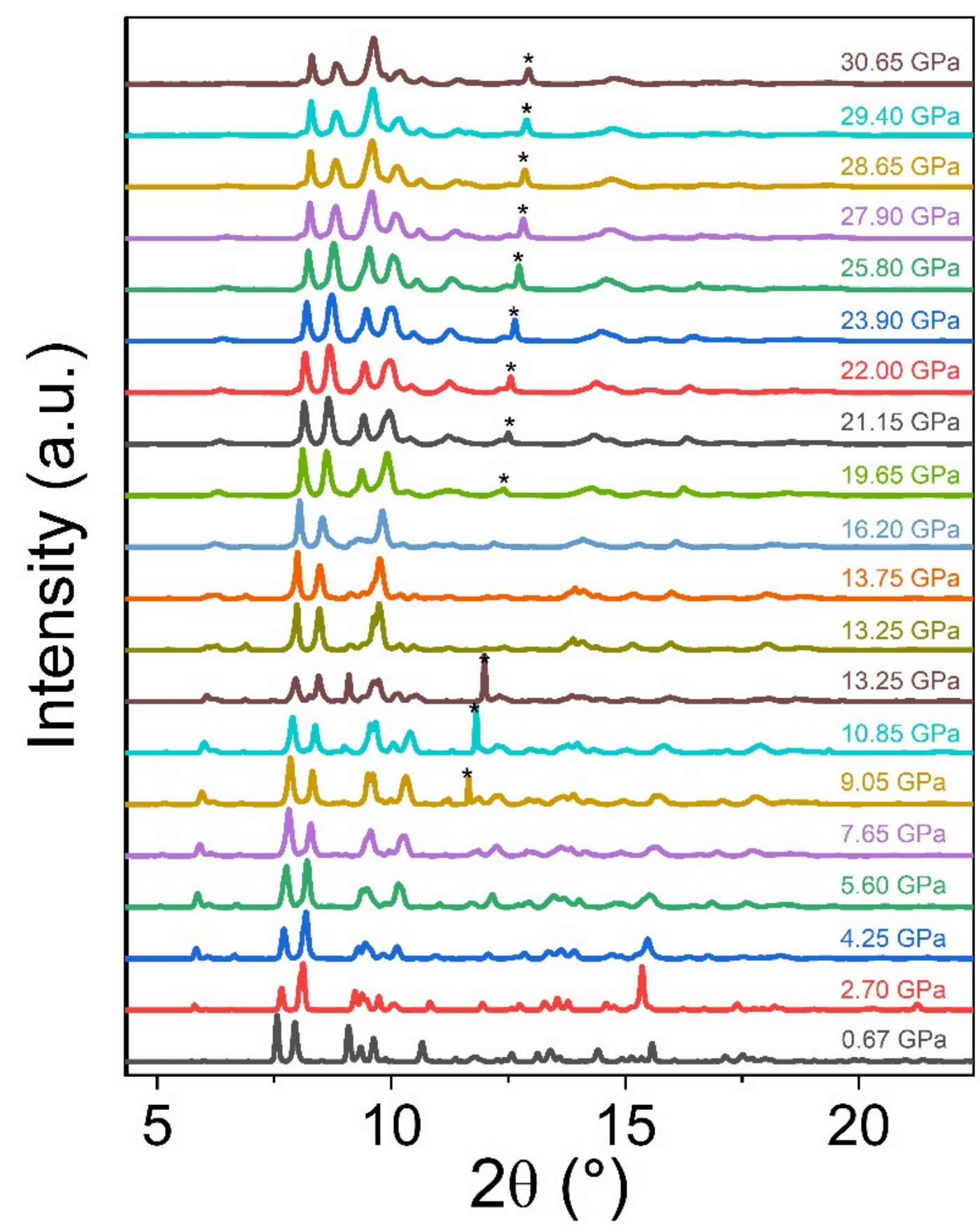

Fig. S8. All diffraction patterns of $LaCu_xTe_2$ collected in a diamond-anvil cell with pressure below 30.65 GPa. Marked additional peaks arise from solidified Ne. At 13.25 GPa, a second pattern was taken at a different sample position to get better diffraction intensity from samples.

Table S1. Fractional Atomic Coordinates and Equivalent Isotropic Displacement Parameters (U_{eq})[a] for $LaCu_xTe_2$ near room temperature (T = 293.16 K).

Atom	*x*	*y*	*z*	U_{eq}/Å^2
La1	0.26189(8)	0.44062(7)	1/4	0.0163(2)
Cu1	0.0891(7)	0.7963(6)	0.0689(11)	0.0342(19)
Te1	0.11733(8)	0.09207(7)	1/4	0.0142(2)
Te2	0.41819(8)	3/4	0	0.0165(2)

[a]U_{eq} is defined as one-third of the trace of the orthogonalized U_{ij} tensor.

Table S2. Anisotropic Displacement Parameters (Å^2×10^3) for $LaCu_xTe_2$. The Anisotropic displacement factor exponent takes the form: $-2\pi^2[h^2a^{*2}U_{11}+2hka^*b^*U_{12}+\ldots]$.

Atom	U_{11}	U_{22}	U_{33}	U_{23}	U_{13}	U_{12}
La1	20.6(3)	15.4(3)	12.9(3)	0	0	1.65(19)
Cu1	23(3)	26(3)	53(4)	17(2)	13(2)	2.8(17)
Te1	15.5(4)	16.9(4)	10.3(3)	0	0	-2.5(2)
Te2	12.8(3)	15.5(3)	21.1(4)	1.6(2)	0	0

Table S3 Bond Lengths for $LaCu_xTe_2$.

Atom	Atom	Length/Å	Atom	Atom	Length/Å
La1	Cu1[1]	3.189(5)	La1	Te2	3.3167(7)
La1	Cu1[2]	3.306(5)	La1	Te2[10]	3.3547(8)
La1	Cu1[3]	3.189(5)	Cu1	Cu1[4]	1.179(13)
La1	Cu1[4]	3.306(5)	Cu1	Cu1[9]	2.286(14)
La1	Te1	3.1939(10)	Cu1	Te1[11]	2.739(6)
La1	Te1[5]	3.3581(7)	Cu1	Te1[12]	2.793(6)
La1	Te1[6]	3.3581(7)	Cu1	Te1[7]	2.630(5)
La1	Te1[7]	3.2003(10)	Cu1	Te2	2.605(5)
La1	Te2[8]	3.3547(8)	Te2	Te2[13]	3.1553(6)
La1	Te2[9]	3.3167(7)	Te2	Te2[9]	3.1553(6)

[1]-X,-1/2+Y,+Z; [2]+X,3/2-Y,1/2+Z; [3]-X,-1/2+Y,1/2-Z; [4]+X,3/2-Y,-Z; [5]+X,1/2-Y,-1/2+Z; [6]+X,1/2-Y,1/2+Z; [7]-X,1/2+Y,1/2-Z; [8]1-X,1-Y,1/2+Z; [9]+X,+Y,1/2-Z; [10]1-X,1-Y,-Z; [11]-X,1-Y,-Z; [12]+X,1+Y,+Z; [13]+X,+Y,-1/2-Z

Table S4 Bond Angles for $LaCu_xTe_2$.

Atom	Atom	Atom	Angle/°	Atom	Atom	Atom	Angle/°
Cu1[1]	La1	Cu1[2]	42.0(2)	Te1[13]	Cu1	La1[11]	63.12(12)

Table S4 Bond Angles for $LaCu_xTe_2$.

Atom	Atom	Atom	Angle/°
Cu1^{1}	La1	Cu1^{3}	72.86(15)
Cu1^{4}	La1	Cu1^{3}	75.0(2)
Cu1^{2}	La1	Cu1^{4}	72.86(15)
Cu1^{1}	La1	Cu1^{4}	98.14(11)
Cu1^{2}	La1	Cu1^{3}	98.14(11)
Cu1^{2}	La1	Te1^{5}	51.85(11)
Cu1^{1}	La1	Te1^{6}	91.29(12)
Cu1^{3}	La1	Te1^{7}	49.56(12)
Cu1^{2}	La1	Te1^{6}	49.38(12)
Cu1^{4}	La1	Te1^{6}	49.56(12)
Cu1^{3}	La1	Te1^{6}	119.70(12)
Cu1^{2}	La1	Te1	48.67(10)
Cu1^{2}	La1	Te1^{7}	91.29(12)
Cu1^{4}	La1	Te1^{7}	119.70(12)
Cu1^{1}	La1	Te1	48.67(10)
Cu1^{1}	La1	Te1^{5}	51.85(11)
Cu1^{1}	La1	Te1^{7}	49.38(12)
Cu1^{1}	La1	Te2^{8}	127.10(10)
Cu1^{1}	La1	Te2^{9}	142.20(11)
Cu1^{4}	La1	Te2^{9}	46.33(9)
Cu1^{2}	La1	Te2^{10}	127.10(10)
Cu1^{4}	La1	Te2^{8}	110.09(12)
Cu1^{2}	La1	Te2	142.21(11)
Cu1^{3}	La1	Te2^{8}	156.36(9)
Cu1^{3}	La1	Te2^{9}	83.61(11)
Cu1^{3}	La1	Te2^{10}	110.09(12)
Cu1^{4}	La1	Te2^{10}	156.36(9)
Cu1^{4}	La1	Te2	83.61(11)
Cu1^{3}	La1	Te2	46.33(9)
Cu1^{1}	La1	Te2	116.70(10)
Cu1^{1}	La1	Te2^{10}	105.43(12)
Cu1^{2}	La1	Te2^{9}	116.70(10)
Cu1^{2}	La1	Te2^{8}	105.43(12)
Te1	La1	Cu1^{4}	119.98(10)
Te1^{5}	La1	Cu1^{3}	49.77(10)
Te1	La1	Cu1^{3}	119.98(10)
Te1^{5}	La1	Cu1^{4}	49.77(10)
Te1^{5}	La1	Te1^{7}	74.377(16)
Te1	La1	Te1^{7}	78.804(17)
Te1^{5}	La1	Te1^{6}	74.377(16)
Te1	La1	Te1^{6}	78.804(17)
Te1^{7}	La1	Te1^{6}	139.97(3)
Te1	La1	Te1^{5}	93.55(2)
Te1^{5}	Cu1	La1^{11}	164.6(2)
Te1^{12}	Cu1	La1^{5}	64.29(12)
Te1^{5}	Cu1	La1	60.64(11)
Te1^{13}	Cu1	La1	139.6(2)
Te1^{5}	Cu1	Te1^{13}	101.58(19)
Te1^{5}	Cu1	Te1^{12}	118.4(2)
Te1^{13}	Cu1	Te1^{12}	91.59(16)
Te2	Cu1	La1^{11}	67.06(11)
Te2	Cu1	La1	63.47(11)
Te2	Cu1	La1^{5}	161.1(3)
Te2	Cu1	Te1^{12}	97.55(19)
Te2	Cu1	Te1^{5}	124.1(2)
Te2	Cu1	Te1^{13}	119.8(2)
La1^{2}	Te1	La1^{6}	105.623(16)
La1^{2}	Te1	La1^{7}	105.623(16)
La1	Te1	La1^{6}	87.850(18)
La1	Te1	La1^{7}	87.850(18)
La1	Te1	La1^{2}	134.40(3)
La1^{6}	Te1	La1^{7}	139.97(3)
Cu1^{2}	Te1	La1	65.56(12)
Cu1^{14}	Te1	La1^{2}	63.86(10)
Cu1^{2}	Te1	La1^{7}	131.68(15)
Cu1^{13}	Te1	La1^{7}	62.08(12)
Cu1^{15}	Te1	La1	151.54(11)
Cu1^{16}	Te1	La1	82.82(12)
Cu1^{13}	Te1	La1^{6}	155.85(13)
Cu1^{13}	Te1	La1^{2}	67.11(11)
Cu1^{14}	Te1	La1	151.54(11)
Cu1^{14}	Te1	La1^{6}	64.24(12)
Cu1^{1}	Te1	La1^{7}	81.28(15)
Cu1^{2}	Te1	La1^{2}	73.62(12)
Cu1^{15}	Te1	La1^{2}	63.86(10)
Cu1^{1}	Te1	La1^{6}	131.68(15)
Cu1^{1}	Te1	La1	65.56(12)
Cu1^{16}	Te1	La1^{6}	62.08(12)
Cu1^{16}	Te1	La1^{7}	155.85(13)
Cu1^{15}	Te1	La1^{6}	109.53(13)
Cu1^{15}	Te1	La1^{7}	64.24(12)
Cu1^{1}	Te1	La1^{2}	73.62(12)
Cu1^{2}	Te1	La1^{6}	81.28(15)
Cu1^{13}	Te1	La1	82.82(12)
Cu1^{14}	Te1	La1^{7}	109.53(13)
Cu1^{16}	Te1	La1^{2}	67.11(11)

Table S4 Bond Angles for $LaCu_xTe_2$.

Atom	Atom	Atom	Angle/°	Atom	Atom	Atom	Angle/°
$Te1^{5}$	La1	$Te2^{10}$	150.481(12)	$Cu1^{15}$	Te1	$Cu1^{14}$	48.3(3)
Te1	La1	Te2	151.343(8)	$Cu1^{2}$	Te1	$Cu1^{13}$	74.58(12)
Te1	La1	$Te2^{9}$	151.343(9)	$Cu1^{1}$	Te1	$Cu1^{13}$	25.3(3)
Te1	La1	$Te2^{10}$	78.43(2)	$Cu1^{13}$	Te1	$Cu1^{16}$	94.6(2)
$Te1^{5}$	La1	$Te2^{8}$	150.481(12)	$Cu1^{2}$	Te1	$Cu1^{15}$	137.48(9)
$Te1^{5}$	La1	$Te2^{9}$	90.37(2)	$Cu1^{13}$	Te1	$Cu1^{14}$	124.99(13)
$Te1^{5}$	La1	Te2	90.37(2)	$Cu1^{2}$	Te1	$Cu1^{14}$	112.4(2)
Te1	La1	$Te2^{8}$	78.43(2)	$Cu1^{1}$	Te1	$Cu1^{16}$	74.58(12)
$Te2^{9}$	La1	$Te1^{7}$	129.38(2)	$Cu1^{16}$	Te1	$Cu1^{14}$	88.41(16)
$Te2^{9}$	La1	$Te1^{6}$	74.968(16)	$Cu1^{2}$	Te1	$Cu1^{1}$	51.5(3)
Te2	La1	$Te1^{6}$	129.38(2)	$Cu1^{1}$	Te1	$Cu1^{14}$	137.48(9)
$Te2^{10}$	La1	$Te1^{6}$	130.178(19)	$Cu1^{13}$	Te1	$Cu1^{15}$	88.41(16)
Te2	La1	$Te1^{7}$	74.968(16)	$Cu1^{1}$	Te1	$Cu1^{15}$	112.4(2)
$Te2^{8}$	La1	$Te1^{7}$	130.18(2)	$Cu1^{16}$	Te1	$Cu1^{15}$	124.99(13)
$Te2^{8}$	La1	$Te1^{6}$	76.198(17)	$Cu1^{2}$	Te1	$Cu1^{16}$	25.3(3)
$Te2^{10}$	La1	$Te1^{7}$	76.198(17)	$La1^{11}$	Te2	$La1^{10}$	115.746(15)
$Te2^{9}$	La1	$Te2^{8}$	84.257(17)	La1	Te2	$La1^{17}$	115.746(15)
Te2	La1	$Te2^{10}$	84.257(17)	$La1^{11}$	Te2	$La1^{17}$	95.743(17)
Te2	La1	$Te2^{8}$	110.32(2)	$La1^{17}$	Te2	$La1^{10}$	85.32(3)
$Te2^{9}$	La1	Te2	56.806(17)	La1	Te2	$La1^{11}$	137.38(3)
$Te2^{8}$	La1	$Te2^{10}$	56.105(18)	La1	Te2	$La1^{10}$	95.743(16)
$Te2^{9}$	La1	$Te2^{10}$	110.32(2)	Cu1	Te2	$La1^{11}$	66.61(12)
$La1^{5}$	Cu1	La1	122.79(16)	Cu1	Te2	$La1^{17}$	124.28(15)
$La1^{5}$	Cu1	$La1^{11}$	107.14(15)	$Cu1^{3}$	Te2	$La1^{10}$	124.28(15)
$La1^{11}$	Cu1	La1	129.62(15)	$Cu1^{3}$	Te2	La1	66.61(12)
$Cu1^{9}$	Cu1	$La1^{5}$	69.00(12)	Cu1	Te2	$La1^{10}$	150.38(15)
$Cu1^{3}$	Cu1	$La1^{11}$	90.7(5)	$Cu1^{3}$	Te2	$La1^{11}$	71.88(12)
$Cu1^{3}$	Cu1	La1	69.8(5)	$Cu1^{3}$	Te2	$La1^{17}$	150.38(15)
$Cu1^{9}$	Cu1	$La1^{11}$	127.50(12)	Cu1	Te2	La1	71.88(12)
$Cu1^{3}$	Cu1	$La1^{5}$	121.78(13)	Cu1	Te2	$Cu1^{3}$	26.2(3)
$Cu1^{9}$	Cu1	La1	71.07(11)	Cu1	Te2	$Te2^{9}$	80.39(15)
$Cu1^{3}$	Cu1	$Cu1^{9}$	137.5(5)	Cu1	Te2	$Te2^{18}$	99.61(15)
$Cu1^{3}$	Cu1	$Te1^{5}$	82.5(4)	$Cu1^{3}$	Te2	$Te2^{18}$	80.39(15)
$Cu1^{9}$	Cu1	$Te1^{5}$	64.25(15)	$Cu1^{3}$	Te2	$Te2^{9}$	99.61(15)
$Cu1^{9}$	Cu1	$Te1^{12}$	65.85(13)	$Te2^{18}$	Te2	La1	118.403(8)
$Cu1^{3}$	Cu1	$Te1^{13}$	72.2(4)	$Te2^{9}$	Te2	La1	61.597(8)
$Cu1^{9}$	Cu1	$Te1^{13}$	137.28(12)	$Te2^{18}$	Te2	$La1^{10}$	61.948(9)
$Cu1^{3}$	Cu1	$Te1^{12}$	156.3(6)	$Te2^{9}$	Te2	$La1^{17}$	61.948(9)
$Cu1^{3}$	Cu1	Te2	76.92(15)	$Te2^{18}$	Te2	$La1^{17}$	118.053(9)
$Cu1^{9}$	Cu1	Te2	99.61(15)	$Te2^{9}$	Te2	$La1^{10}$	118.053(9)
$Te1^{12}$	Cu1	$La1^{11}$	66.20(11)	$Te2^{18}$	Te2	$La1^{11}$	61.597(8)
$Te1^{13}$	Cu1	$La1^{5}$	68.53(12)	$Te2^{9}$	Te2	$La1^{11}$	118.403(8)
$Te1^{12}$	Cu1	La1	128.8(2)	$Te2^{9}$	Te2	$Te2^{18}$	180.0

Table S4 Bond Angles for $LaCu_xTe_2$.

Atom	Atom	Atom	Angle/°	Atom	Atom	Atom	Angle/°
$Te1^5$	Cu1	$La1^5$	65.77(11)				

^{1}-X,-1/2+Y,+Z; ^{2}-X,-1/2+Y,1/2-Z; ^{3}+X,3/2-Y,-Z; ^{4}+X,3/2-Y,1/2+Z; ^{5}-X,1/2+Y,1/2-Z; ^{6}+X,1/2-Y,1/2+Z; ^{7}+X,1/2-Y,-1/2+Z; ^{8}1-X,1-Y,1/2+Z; ^{9}+X,+Y,1/2-Z; ^{10}1-X,1-Y,-Z; ^{11}+X,3/2-Y,-1/2+Z; ^{12}+X,1+Y,+Z; ^{13}-X,1-Y,-Z; ^{14}+X,-1+Y,1/2-Z; ^{15}+X,-1+Y,+Z; ^{16}-X,1-Y,1/2+Z; ^{17}1-X,1/2+Y,1/2-Z; ^{18}+X,+Y,-1/2-Z

Table S5 Atomic Occupancy for $LaCu_xTe_2$.

**Atom**	***Occupancy***
Cu1	0.204(5)

Table S6. DFT simulated formation energy and stability of different phases in the La-Cu-Te system.

Composition	Space group	Formation Energy (eV/atom)	Energy above hull (eV/atom)	Relevant interpretations
La_3CuTe_5	*Pnma*	-1.363	0.000	Most stable compound in the system. Reported in literatures.[13]
La_4CuTe_8	*P*1	-1.281	0.014	Triclinic space group was used due to the absence of symmetry in the copper occupations.
La_2CuTe_4	$P2_1$	-1.186	0.016	Monoclinic space group was used due to the absence of symmetry in the copper occupations.
La_2CuTe_4	*Pc*	-1.182	0.019	Monoclinic space group was used due to the absence of symmetry in the copper occupations.
$La_3Cu_{5-x}Te_7$	*Pnma*	-	-	A competing phase in the La-Cu-Te system. Simply assigning Cu sites as either fully occupied or vacant produces either unrealistically short Cu-Cu separations or wrong composition. Formation energy unavailable.
La_2CuTe_3	*Pnma*	-1.199	0.106	-
$LaCu_2Te_4$	*C*2/*c*	-0.521	0.125	-
La_3CuTe_5	*Pnma*	-1.163	0.200	$CsBi_3Se_5$ prototype was employed.
La_2CuTe	$Fm\bar{3}m$	-0.639	0.246	FullHeusler Cu_2MnAl prototype was employed
LaCuTe	$F\bar{4}3m$	-0.660	0.402	HalfHeusler MgAgAs prototype was employed
$LaCuTe_2$	$Fm\bar{3}m$	-0.629	0.423	FullHeusler Cu_2MnAl prototype was employed
LaCuTe	$F\bar{4}3m$	-0.552	0.509	HalfHeusler MgAgAs prototype was employed
$LaCu_2Te$	$Fm\bar{3}m$	-0.265	0.531	FullHeusler Cu_2MnAl prototype was employed
LaCuTe	$F\bar{4}3m$	-0.286	0.776	HalfHeusler MgAgAs prototype was employed

Table S7. Atomic coordinates (x10^4), occupancy and equivalent isotropic displacement parameters (Å^{2}x10^3) for $LaCu_{0.347}Te_2$ at 100.03 K with estimated standard deviations in parentheses.

Label	x	y	z	Occupancy	U_{eq}^*
La(1)	7349(1)	7500	583(1)	1	7(1)
La(2)	7421(1)	2500	4388(1)	1	5(1)
Te(1)	8816(1)	2500	878(1)	1	5(1)
Te(2)	8822(1)	7500	4048(1)	1	5(1)
Te(3)	4174(1)	9(1)	2527(1)	1	6(1)
Cu(1)	862	940	2983.61	0.084(3)	11(2)
Cu(2)	950.82	-479.55	1992.39	0.259(3)	5(1)

$^*U_{eq}$ is defined as one third of the trace of the orthogonalized U_{ij} tensor.

Table S8. Anisotropic displacement parameters (Å^{2}x10^3) for $LaCu_{0.347}Te_2$ at 100.03 K with estimated standard deviations in parentheses.

Label	U_{11}	U_{22}	U_{33}	U_{12}	U_{13}	U_{23}
La(1)	11(1)	5(1)	6(1)	0	1(1)	0
La(2)	8(1)	3(1)	5(1)	0	-2(1)	0
Te(1)	7(1)	1(1)	7(1)	0	0(1)	0
Te(2)	5(1)	2(1)	6(1)	0	-1(1)	0
Te(3)	5(1)	7(1)	6(1)	-1(1)	0(1)	1(1)

Table S9. Fourier components of the displacive modulation for $LaCu_{0.347}Te_2$ at 100.03 K with estimated standard deviations in parentheses.

Label	Vector	Axis	cos displacement	sin displacement
La(1)	q(1)	x	0.002202(10)	-0.01189(5)
La(1)	q(1)	y	0.01013(6)	0.001877(10)
La(1)	q(1)	z	0.000141(7)	-0.00076(4)
La(2)	q(1)	x	-0.00221(5)	0.000430(9)
La(2)	q(1)	y	-0.000533(10)	-0.00274(5)
La(2)	q(1)	z	-0.00187(3)	0.000364(7)
Te(1)	q(1)	x	0.001431(14)	-0.00505(5)
Te(1)	q(1)	y	0.00279(5)	0.000790(15)
Te(1)	q(1)	z	-0.000793(10)	0.00280(4)
Te(2)	q(1)	x	0.00198(5)	-0.000612(14)
Te(2)	q(1)	y	-0.000958(16)	-0.00310(5)
Te(2)	q(1)	z	0.00205(4)	-0.000632(11)
Te(3)	q(1)	x	0.00265(4)	-0.00339(4)

Te(3)	q(1)	y	-0.01030(5)	0.00963(5)
Te(3)	q(1)	z	-0.00071(3)	0.00121(3)
Cu(1)	q(1)	x	0.0014(9)	-0.0032(9)
Cu(1)	q(1)	y	-0.0044(10)	0.0102(10)
Cu(1)	q(1)	z	-0.0014(7)	0.0029(7)
Cu(2)	q(1)	x	0.0061(2)	0.0011(3)
Cu(2)	q(1)	y	0.0162(3)	0.0111(3)
Cu(2)	q(1)	z	-0.00273(19)	-0.00143(19)

Table S10. Bond lengths distributions [Å] for $LaCu_{0.347}Te_2$ at 100.03 K with estimated standard deviations in parentheses.

Label	Average Distance	Minimum Distance	Maximum Distance
La(1)-Te(1)	3.3460(4)	3.2798(5)	3.4114(5)
La(1)-Te(1)#1	3.3462(4)	3.2798(4)	3.4114(4)
La(1)-Te(1)#2	3.2114(8)	3.1747(9)	3.2468(9)
La(1)-Te(2)	3.1833(8)	3.1666(8)	3.1994(8)
La(1)-Te(3)#1	3.3561(7)	3.3421(7)	3.3703(7)
La(1)-Te(3)#3	3.3088(7)	3.3028(7)	3.3138(7)
La(1)-Te(3)#4	3.3089(7)	3.3028(7)	3.3138(7)
La(1)-Te(3)#5	3.3562(7)	3.3421(7)	3.3703(7)
La(1)-Cu(2)#6	3.280(2)	3.233(2)	3.326(2)
La(1)-Cu(2)#3	3.1821(18)	3.0227(19)	3.3420(19)
La(1)-Cu(2)#4	3.1826(18)	3.0227(19)	3.3419(19)
La(1)-Cu(2)#7	3.280(2)	3.233(2)	3.326(2)
La(2)-Te(1)	3.2009(8)	3.1736(8)	3.2287(8)
La(2)-Te(2)#8	3.3324(4)	3.3191(4)	3.3458(4)
La(2)-Te(2)	3.3325(4)	3.3191(4)	3.3458(4)
La(2)-Te(2)#9	3.1869(8)	3.1865(9)	3.1872(9)
La(2)-Te(3)	3.3530(7)	3.3450(7)	3.3597(7)
La(2)-Te(3)#10	3.3199(7)	3.3001(7)	3.3406(7)
La(2)-Te(3)#11	3.3196(7)	3.3001(7)	3.3406(7)
La(2)-Te(3)#5	3.3529(7)	3.3450(7)	3.3597(7)
La(2)-Cu(1)#12	3.073(7)	3.008(7)	3.139(7)
La(2)-Cu(1)#7	3.073(7)	3.008(7)	3.139(7)
Te(1)-Cu(1)#12	2.589(6)	2.573(6)	2.606(6)
Te(1)-Cu(1)#7	2.589(6)	2.573(6)	2.606(6)

Te(1)-Cu(2)#12	2.6678(19)	2.620(2)	2.719(2)
Te(1)-Cu(2)#3	2.7799(18)	2.7300(18)	2.8323(18)
Te(1)-Cu(2)#13	2.7806(18)	2.7300(18)	2.8323(18)
Te(1)-Cu(2)#7	2.6684(19)	2.620(2)	2.719(2)
Te(2)-Cu(1)#6	2.824(7)	2.779(7)	2.870(7)
Te(2)-Cu(1)#10	2.742(6)	2.710(6)	2.775(6)
Te(2)-Cu(1)#14	2.742(6)	2.710(6)	2.775(6)
Te(2)-Cu(1)#7	2.824(7)	2.779(7)	2.870(7)
Te(2)-Cu(2)#6	2.7240(19)	2.6150(19)	2.8362(19)
Te(2)-Cu(2)#7	2.7243(19)	2.6150(19)	2.8362(19)
Te(3)-Cu(1)	2.647(7)	2.614(7)	2.679(7)
Te(3)-Cu(2)	2.543(2)	2.486(2)	2.603(2)
Cu(1)-Cu(1)#5	1.958(9)	1.821(9)	2.097(9)
Cu(1)-Cu(2)	1.236(6)	1.187(6)	1.284(6)
Cu(2)-Cu(2)#15	2.538(3)	2.419(3)	2.654(3)

Symmetry transformations used to generate equivalent atoms:
(1) x1,x2+1,x3,x4 (2) -x1+2,x2+1/2,-x3,-x3+x4+1/2 (3) -x1+1,x2+1/2,-x3,-x3+x4+1/2 (4) -x1+1,-x2+1,-x3,-x4 (5) x1,-x2+1/2,x3,x3-x4+1/2 (6) x1+1,x2+1,x3,x4 (7) x1+1,-x2+1/2,x3,x3-x4+1/2 (8) x1,x2-1,x3,x4 (9) -x1+2,x2-1/2,-x3+1,-x3+x4+1/2 (10) -x1+1,x2+1/2,-x3+1,-x3+x4+1/2 (11) -x1+1,-x2,-x3+1,-x4 (12) x1+1,x2,x3,x4 (13) -x1+1,-x2,-x3,-x4 (14) -x1+1,-x2+1,-x3+1,-x4 (15) x1,-x2-1/2,x3,x3-x4+1/2 (16) -x1+2,x2-1/2,-x3,-x3+x4+1/2 (17) -x1+2,x2+1/2,-x3+1,-x3+x4+1/2 (18) -x1+1,x2-1/2,-x3,-x3+x4+1/2 (19) -x1+1,x2-1/2,-x3+1,-x3+x4+1/2 (20) x1-1,x2,x3,x4 (21) x1-1,x2-1,x3,x4

Table S11. Bond angles distributions [°] for $LaCu_{0.347}Te_2$ at 100.03 K with estimated standard deviations in parentheses.

Label	Average Angle	Minimun Angle	Maximum Angle
Te(1)-La(1)-Te(1)#1	139.52(2)	136.82(2)	142.28(2)
Te(1)-La(1)-Te(1)#2	73.681(14)	71.429(13)	75.999(14)
Te(1)-La(1)-Te(2)	78.990(15)	77.442(15)	80.575(15)
Te(1)-La(1)-Te(3)#1	130.432(19)	128.509(19)	132.27(2)
Te(1)-La(1)-Te(3)#3	74.730(14)	73.796(13)	75.655(13)
Te(1)-La(1)-Te(3)#4	128.603(19)	126.872(19)	130.25(2)
Te(1)-La(1)-Te(3)#5	76.562(14)	75.912(13)	77.223(13)
Te(1)-La(1)-Cu(2)#6	92.84(4)	90.14(4)	95.60(4)
Te(1)-La(1)-Cu(2)#3	50.37(3)	47.71(3)	53.17(4)
Te(1)-La(1)-Cu(2)#4	117.68(4)	115.45(4)	119.98(4)
Te(1)-La(1)-Cu(2)#7	47.47(3)	46.88(3)	48.08(3)

Te(1)#1-La(1)-Te(1)#2	73.675(14)	71.429(13)	75.999(14)
Te(1)#1-La(1)-Te(2)	78.986(15)	77.442(14)	80.575(15)
Te(1)#1-La(1)-Te(3)#1	76.561(14)	75.913(13)	77.223(13)
Te(1)#1-La(1)-Te(3)#3	128.601(19)	126.873(19)	130.25(2)
Te(1)#1-La(1)-Te(3)#4	74.728(14)	73.796(13)	75.656(14)
Te(1)#1-La(1)-Te(3)#5	130.432(19)	128.510(19)	132.27(2)
Te(1)#1-La(1)-Cu(2)#6	47.47(3)	46.88(3)	48.08(3)
Te(1)#1-La(1)-Cu(2)#3	117.67(4)	115.45(4)	119.98(4)
Te(1)#1-La(1)-Cu(2)#4	50.36(3)	47.71(3)	53.17(4)
Te(1)#1-La(1)-Cu(2)#7	92.84(4)	90.14(4)	95.60(4)
Te(1)#2-La(1)-Te(2)	92.19(2)	89.98(2)	94.50(2)
Te(1)#2-La(1)-Te(3)#1	149.951(14)	147.246(14)	152.731(14)
Te(1)#2-La(1)-Te(3)#3	90.536(18)	89.579(17)	91.501(17)
Te(1)#2-La(1)-Te(3)#4	90.533(18)	89.579(17)	91.502(17)
Te(1)#2-La(1)-Te(3)#5	149.958(14)	147.246(13)	152.730(12)
Te(1)#2-La(1)-Cu(2)#6	50.70(3)	49.38(3)	52.05(3)
Te(1)#2-La(1)-Cu(2)#3	49.31(4)	48.82(4)	49.83(4)
Te(1)#2-La(1)-Cu(2)#4	49.31(4)	48.82(4)	49.83(4)
Te(1)#2-La(1)-Cu(2)#7	50.70(3)	49.38(3)	52.05(3)
Te(2)-La(1)-Te(3)#1	78.081(17)	77.174(16)	79.021(16)
Te(2)-La(1)-Te(3)#3	151.591(12)	149.482(12)	153.728(13)
Te(2)-La(1)-Te(3)#4	151.585(12)	149.482(13)	153.728(14)
Te(2)-La(1)-Te(3)#5	78.084(17)	77.174(16)	79.021(16)
Te(2)-La(1)-Cu(2)#6	49.83(3)	47.66(3)	52.08(3)
Te(2)-La(1)-Cu(2)#3	120.24(4)	116.85(4)	123.84(4)
Te(2)-La(1)-Cu(2)#4	120.24(4)	116.85(3)	123.84(4)
Te(2)-La(1)-Cu(2)#7	49.84(3)	47.66(3)	52.08(3)
Te(3)#1-La(1)-Te(3)#3	111.501(19)	108.78(2)	114.18(2)
Te(3)#1-La(1)-Te(3)#4	85.507(16)	85.436(16)	85.592(15)
Te(3)#1-La(1)-Te(3)#5	56.019(13)	52.959(14)	59.017(15)
Te(3)#1-La(1)-Cu(2)#6	104.54(3)	103.75(3)	105.36(3)
Te(3)#1-La(1)-Cu(2)#3	157.35(4)	154.50(4)	160.24(4)
Te(3)#1-La(1)-Cu(2)#4	111.76(4)	109.67(3)	113.92(4)
Te(3)#1-La(1)-Cu(2)#7	127.86(3)	126.61(4)	129.10(4)
Te(3)#3-La(1)-Te(3)#4	56.460(14)	53.522(14)	59.335(15)
Te(3)#3-La(1)-Te(3)#5	85.507(16)	85.436(16)	85.592(15)

Te(3)#3-La(1)-Cu(2)#6	141.14(4)	139.75(4)	142.54(4)
Te(3)#3-La(1)-Cu(2)#3	46.06(4)	45.92(4)	46.20(4)
Te(3)#3-La(1)-Cu(2)#4	82.06(4)	79.41(4)	84.58(3)
Te(3)#3-La(1)-Cu(2)#7	114.42(3)	113.62(3)	115.23(3)
Te(3)#4-La(1)-Te(3)#5	111.501(19)	108.78(2)	114.18(2)
Te(3)#4-La(1)-Cu(2)#6	114.42(3)	113.62(3)	115.23(3)
Te(3)#4-La(1)-Cu(2)#3	82.05(4)	79.41(4)	84.58(3)
Te(3)#4-La(1)-Cu(2)#4	46.06(4)	45.92(4)	46.20(4)
Te(3)#4-La(1)-Cu(2)#7	141.14(4)	139.75(4)	142.54(4)
Te(3)#5-La(1)-Cu(2)#6	127.85(3)	126.61(3)	129.10(3)
Te(3)#5-La(1)-Cu(2)#3	111.76(4)	109.67(3)	113.92(4)
Te(3)#5-La(1)-Cu(2)#4	157.35(4)	154.50(4)	160.24(4)
Te(3)#5-La(1)-Cu(2)#7	104.54(3)	103.75(3)	105.36(3)
Cu(2)#6-La(1)-Cu(2)#3	97.78(5)	95.72(5)	99.88(5)
Cu(2)#6-La(1)-Cu(2)#4	71.40(5)	70.45(5)	72.41(5)
Cu(2)#6-La(1)-Cu(2)#7	45.50(5)	42.84(5)	48.24(5)
Cu(2)#3-La(1)-Cu(2)#4	71.97(5)	71.04(5)	72.98(5)
Cu(2)#3-La(1)-Cu(2)#7	71.40(5)	70.45(5)	72.41(5)
Cu(2)#4-La(1)-Cu(2)#7	97.78(5)	95.72(5)	99.88(5)
Te(1)-La(2)-Te(2)#8	78.950(14)	78.157(14)	79.735(14)
Te(1)-La(2)-Te(2)	78.937(14)	78.157(14)	79.734(15)
Te(1)-La(2)-Te(2)#9	95.24(2)	94.28(2)	96.21(2)
Te(1)-La(2)-Te(3)	78.591(17)	77.958(18)	79.214(17)
Te(1)-La(2)-Te(3)#10	151.200(12)	148.988(13)	153.476(13)
Te(1)-La(2)-Te(3)#11	151.228(12)	148.988(12)	153.476(12)
Te(1)-La(2)-Te(3)#5	78.588(17)	77.959(18)	79.214(17)
Te(1)-La(2)-Cu(1)#12	48.69(11)	48.26(11)	49.12(11)
Te(1)-La(2)-Cu(1)#7	48.69(11)	48.26(11)	49.12(11)
Te(2)#8-La(2)-Te(2)	140.79(2)	140.02(2)	141.53(2)
Te(2)#8-La(2)-Te(2)#9	75.125(15)	74.362(15)	75.885(15)
Te(2)#8-La(2)-Te(3)	76.113(13)	75.217(12)	77.005(13)
Te(2)#8-La(2)-Te(3)#10	129.601(19)	127.866(18)	131.307(18)
Te(2)#8-La(2)-Te(3)#11	75.130(13)	73.521(13)	76.741(13)
Te(2)#8-La(2)-Te(3)#5	129.703(19)	127.848(19)	131.578(19)
Te(2)#8-La(2)-Cu(1)#12	52.14(12)	50.85(12)	53.42(12)
Te(2)#8-La(2)-Cu(1)#7	89.21(12)	88.47(12)	89.92(12)

Te(2)-La(2)-Te(2)#9	75.132(15)	74.361(15)	75.886(15)
Te(2)-La(2)-Te(3)	129.704(19)	127.848(19)	131.578(19)
Te(2)-La(2)-Te(3)#10	75.112(13)	73.521(14)	76.741(14)
Te(2)-La(2)-Te(3)#11	129.588(19)	127.866(19)	131.308(19)
Te(2)-La(2)-Te(3)#5	76.117(13)	75.217(13)	77.005(13)
Te(2)-La(2)-Cu(1)#12	89.20(12)	88.47(12)	89.92(12)
Te(2)-La(2)-Cu(1)#7	52.13(12)	50.85(12)	53.42(12)
Te(2)#9-La(2)-Te(3)	151.231(12)	149.598(11)	152.858(11)
Te(2)#9-La(2)-Te(3)#10	90.029(17)	89.781(17)	90.283(17)
Te(2)#9-La(2)-Te(3)#11	90.034(17)	89.781(17)	90.283(17)
Te(2)#9-La(2)-Te(3)#5	151.242(12)	149.598(12)	152.858(11)
Te(2)#9-La(2)-Cu(1)#12	51.90(11)	51.73(11)	52.08(11)
Te(2)#9-La(2)-Cu(1)#7	51.91(11)	51.73(11)	52.08(11)
Te(3)-La(2)-Te(3)#10	108.956(19)	107.812(19)	110.126(19)
Te(3)-La(2)-Te(3)#11	83.260(15)	82.926(16)	83.648(16)
Te(3)-La(2)-Te(3)#5	55.608(13)	52.753(14)	58.498(14)
Te(3)-La(2)-Cu(1)#12	107.88(12)	106.29(12)	109.45(12)
Te(3)-La(2)-Cu(1)#7	127.19(11)	126.98(11)	127.39(11)
Te(3)#10-La(2)-Te(3)#11	56.665(14)	53.956(14)	59.342(14)
Te(3)#10-La(2)-Te(3)#5	83.258(15)	82.926(15)	83.648(15)
Te(3)#10-La(2)-Cu(1)#12	141.66(12)	140.98(12)	142.30(11)
Te(3)#10-La(2)-Cu(1)#7	118.85(11)	117.50(11)	120.19(11)
Te(3)#11-La(2)-Te(3)#5	108.938(19)	107.811(18)	110.126(18)
Te(3)#11-La(2)-Cu(1)#12	118.87(11)	117.50(11)	120.19(11)
Te(3)#11-La(2)-Cu(1)#7	141.67(12)	140.98(12)	142.30(11)
Te(3)#5-La(2)-Cu(1)#12	127.19(11)	126.98(11)	127.39(11)
Te(3)#5-La(2)-Cu(1)#7	107.89(12)	106.29(12)	109.45(12)
Cu(1)#12-La(2)-Cu(1)#7	37.14(17)	35.22(17)	39.04(17)
La(1)#8-Te(1)-La(1)	139.54(2)	138.74(2)	140.30(2)
La(1)#8-Te(1)-La(1)#16	106.273(15)	103.660(15)	108.925(15)
La(1)#8-Te(1)-La(2)	87.590(16)	86.819(15)	88.378(16)
La(1)#8-Te(1)-Cu(1)#12	84.44(14)	82.05(14)	86.84(14)
La(1)#8-Te(1)-Cu(1)#7	127.78(14)	126.58(14)	128.98(14)
La(1)#8-Te(1)-Cu(2)#12	65.00(4)	62.75(4)	67.24(4)
La(1)#8-Te(1)-Cu(2)#3	112.48(4)	110.46(4)	114.46(4)
La(1)#8-Te(1)-Cu(2)#13	61.73(4)	58.64(4)	64.92(4)

La(1)#8-Te(1)-Cu(2)#7	153.23(4)	152.47(5)	153.92(4)
La(1)-Te(1)-La(1)#16	106.316(15)	103.661(16)	108.925(16)
La(1)-Te(1)-La(2)	87.594(16)	86.819(15)	88.378(16)
La(1)-Te(1)-Cu(1)#12	127.80(14)	126.58(14)	128.99(14)
La(1)-Te(1)-Cu(1)#7	84.46(14)	82.06(14)	86.84(14)
La(1)-Te(1)-Cu(2)#12	153.22(4)	152.47(5)	153.92(4)
La(1)-Te(1)-Cu(2)#3	61.78(4)	58.64(4)	64.92(4)
La(1)-Te(1)-Cu(2)#13	112.51(4)	110.46(4)	114.46(4)
La(1)-Te(1)-Cu(2)#7	64.99(4)	62.75(4)	67.24(4)
La(1)#16-Te(1)-La(2)	132.68(2)	132.65(2)	132.74(2)
La(1)#16-Te(1)-Cu(1)#12	73.37(15)	72.73(15)	73.99(15)
La(1)#16-Te(1)-Cu(1)#7	73.38(15)	72.73(15)	73.99(15)
La(1)#16-Te(1)-Cu(2)#12	64.71(4)	61.73(4)	67.75(4)
La(1)#16-Te(1)-Cu(2)#3	65.91(4)	65.23(4)	66.64(4)
La(1)#16-Te(1)-Cu(2)#13	65.92(4)	65.23(4)	66.64(4)
La(1)#16-Te(1)-Cu(2)#7	64.76(4)	61.73(4)	67.75(4)
La(2)-Te(1)-Cu(1)#12	63.07(14)	62.06(14)	64.09(14)
La(2)-Te(1)-Cu(1)#7	63.07(14)	62.06(14)	64.09(14)
La(2)-Te(1)-Cu(2)#12	82.54(4)	82.04(4)	83.02(4)
La(2)-Te(1)-Cu(2)#3	149.08(4)	146.00(4)	152.13(4)
La(2)-Te(1)-Cu(2)#13	149.03(4)	146.00(4)	152.13(4)
La(2)-Te(1)-Cu(2)#7	82.53(4)	82.04(4)	83.02(4)
Cu(1)#12-Te(1)-Cu(1)#7	44.4(2)	41.4(2)	47.5(2)
Cu(1)#12-Te(1)-Cu(2)#12	27.15(14)	26.01(14)	28.23(14)
Cu(1)#12-Te(1)-Cu(2)#3	138.69(15)	137.96(16)	139.60(15)
Cu(1)#12-Te(1)-Cu(2)#13	113.99(14)	112.57(14)	115.38(14)
Cu(1)#12-Te(1)-Cu(2)#7	68.93(15)	65.99(15)	71.87(15)
Cu(1)#7-Te(1)-Cu(2)#12	68.91(15)	65.99(15)	71.87(15)
Cu(1)#7-Te(1)-Cu(2)#3	114.01(14)	112.57(14)	115.38(14)
Cu(1)#7-Te(1)-Cu(2)#13	138.70(15)	137.95(15)	139.60(15)
Cu(1)#7-Te(1)-Cu(2)#7	27.16(14)	26.01(14)	28.23(14)
Cu(2)#12-Te(1)-Cu(2)#3	126.63(6)	123.58(6)	129.80(6)
Cu(2)#12-Te(1)-Cu(2)#13	87.59(5)	86.07(5)	89.29(5)
Cu(2)#12-Te(1)-Cu(2)#7	89.02(6)	85.90(6)	92.14(6)
Cu(2)#3-Te(1)-Cu(2)#13	54.30(5)	52.33(6)	56.27(5)
Cu(2)#3-Te(1)-Cu(2)#7	87.62(5)	86.07(5)	89.29(5)

Cu(2)#13-Te(1)-Cu(2)#7	126.68(6)	123.58(6)	129.80(6)
La(1)-Te(2)-La(2)	88.118(15)	87.010(15)	89.216(15)
La(1)-Te(2)-La(2)#1	88.121(15)	87.010(15)	89.216(15)
La(1)-Te(2)-La(2)#17	135.71(2)	134.29(2)	137.12(2)
La(1)-Te(2)-Cu(1)#6	83.97(12)	82.32(12)	85.64(12)
La(1)-Te(2)-Cu(1)#10	154.19(14)	152.20(14)	156.13(14)
La(1)-Te(2)-Cu(1)#14	154.20(14)	152.20(14)	156.13(14)
La(1)-Te(2)-Cu(1)#7	83.97(12)	82.32(12)	85.64(12)
La(1)-Te(2)-Cu(2)#6	66.92(4)	65.75(4)	68.12(4)
La(1)-Te(2)-Cu(2)#7	66.92(4)	65.75(4)	68.12(4)
La(2)-Te(2)-La(2)#1	140.79(2)	140.09(2)	141.46(2)
La(2)-Te(2)-La(2)#17	104.868(15)	104.113(15)	105.640(15)
La(2)-Te(2)-Cu(1)#6	158.30(13)	156.47(13)	160.13(13)
La(2)-Te(2)-Cu(1)#10	67.07(14)	65.68(13)	68.45(14)
La(2)-Te(2)-Cu(1)#14	106.42(13)	104.90(14)	107.95(13)
La(2)-Te(2)-Cu(1)#7	59.21(14)	57.30(13)	61.17(14)
La(2)-Te(2)-Cu(2)#6	133.63(4)	131.50(4)	135.70(4)
La(2)-Te(2)-Cu(2)#7	79.29(4)	77.15(4)	81.50(4)
La(2)#1-Te(2)-La(2)#17	104.870(15)	104.113(16)	105.640(16)
La(2)#1-Te(2)-Cu(1)#6	59.22(14)	57.30(13)	61.17(14)
La(2)#1-Te(2)-Cu(1)#10	106.42(13)	104.90(14)	107.95(13)
La(2)#1-Te(2)-Cu(1)#14	67.07(14)	65.68(13)	68.45(14)
La(2)#1-Te(2)-Cu(1)#7	158.31(13)	156.47(13)	160.13(13)
La(2)#1-Te(2)-Cu(2)#6	79.29(4)	77.15(4)	81.50(4)
La(2)#1-Te(2)-Cu(2)#7	133.64(4)	131.50(4)	135.70(4)
La(2)#17-Te(2)-Cu(1)#6	68.37(13)	67.69(14)	69.04(13)
La(2)#17-Te(2)-Cu(1)#10	61.89(15)	60.69(15)	63.10(15)
La(2)#17-Te(2)-Cu(1)#14	61.89(15)	60.69(15)	63.10(15)
La(2)#17-Te(2)-Cu(1)#7	68.37(13)	67.69(14)	69.04(13)
La(2)#17-Te(2)-Cu(2)#6	74.12(4)	73.86(4)	74.38(4)
La(2)#17-Te(2)-Cu(2)#7	74.12(4)	73.86(4)	74.38(4)
Cu(1)#6-Te(2)-Cu(1)#10	121.68(19)	119.38(19)	124.00(19)
Cu(1)#6-Te(2)-Cu(1)#14	88.81(18)	87.30(18)	90.32(18)
Cu(1)#6-Te(2)-Cu(1)#7	99.79(19)	99.21(19)	100.40(19)
Cu(1)#6-Te(2)-Cu(2)#6	25.67(13)	24.45(13)	26.89(13)
Cu(1)#6-Te(2)-Cu(2)#7	79.04(13)	78.59(13)	79.42(14)

Cu(1)#10-Te(2)-Cu(1)#14	41.82(19)	39.21(19)	44.46(18)
Cu(1)#10-Te(2)-Cu(1)#7	88.81(18)	87.31(18)	90.32(18)
Cu(1)#10-Te(2)-Cu(2)#6	135.70(15)	134.24(16)	137.22(15)
Cu(1)#10-Te(2)-Cu(2)#7	112.56(14)	110.67(14)	114.48(15)
Cu(1)#14-Te(2)-Cu(1)#7	121.67(18)	119.38(19)	124.00(19)
Cu(1)#14-Te(2)-Cu(2)#6	112.57(14)	110.67(14)	114.48(15)
Cu(1)#14-Te(2)-Cu(2)#7	135.70(15)	134.24(16)	137.22(15)
Cu(1)#7-Te(2)-Cu(2)#6	79.04(13)	78.59(13)	79.42(14)
Cu(1)#7-Te(2)-Cu(2)#7	25.67(13)	24.45(13)	26.89(13)
Cu(2)#6-Te(2)-Cu(2)#7	55.41(6)	54.06(6)	56.89(6)
La(1)#8-Te(3)-La(1)#18	94.467(18)	94.339(18)	94.550(18)
La(1)#8-Te(3)-La(2)	84.977(16)	83.640(15)	86.357(15)
La(1)#8-Te(3)-La(2)#19	116.433(16)	114.270(15)	118.566(16)
La(1)#8-Te(3)-Cu(1)	151.69(13)	149.39(13)	153.87(13)
La(1)#8-Te(3)-Cu(2)	124.42(4)	121.03(4)	127.71(4)
La(1)#18-Te(3)-La(2)	115.310(16)	112.584(16)	117.999(16)
La(1)#18-Te(3)-La(2)#19	137.46(2)	136.76(2)	138.15(2)
La(1)#18-Te(3)-Cu(1)	71.01(13)	70.23(12)	71.84(13)
La(1)#18-Te(3)-Cu(2)	64.26(4)	60.97(4)	67.60(4)
La(2)-Te(3)-La(2)#19	96.736(17)	96.297(17)	97.176(17)
La(2)-Te(3)-Cu(1)	122.98(13)	121.22(13)	124.74(13)
La(2)-Te(3)-Cu(2)	150.33(4)	147.25(4)	153.35(4)
La(2)#19-Te(3)-Cu(1)	68.20(13)	67.43(13)	68.98(13)
La(2)#19-Te(3)-Cu(2)	74.04(4)	71.75(4)	76.36(4)
Cu(1)-Te(3)-Cu(2)	27.44(14)	26.19(14)	28.66(14)
La(2)#20-Cu(1)-Te(1)#20	68.24(16)	67.65(16)	68.82(17)
La(2)#20-Cu(1)-Te(2)#21	68.66(15)	67.96(15)	69.31(15)
La(2)#20-Cu(1)-Te(2)#19	66.20(14)	64.89(13)	67.50(14)
La(2)#20-Cu(1)-Te(3)	163.7(2)	162.8(2)	164.5(2)
La(2)#20-Cu(1)-Cu(1)#5	71.4(3)	70.2(3)	72.7(3)
La(2)#20-Cu(1)-Cu(2)	123.4(4)	120.7(4)	126.0(4)
Te(1)#20-Cu(1)-Te(2)#21	100.2(2)	98.9(2)	101.4(2)
Te(1)#20-Cu(1)-Te(2)#19	124.6(2)	121.9(2)	127.2(3)
Te(1)#20-Cu(1)-Te(3)	124.5(2)	124.2(2)	124.7(2)
Te(1)#20-Cu(1)-Cu(1)#5	67.8(3)	66.3(2)	69.3(3)
Te(1)#20-Cu(1)-Cu(2)	80.0(3)	78.2(3)	81.9(3)

Te(2)#21-Cu(1)-Te(2)#19	91.16(18)	90.26(18)	92.10(18)
Te(2)#21-Cu(1)-Te(3)	114.6(2)	112.5(2)	116.7(2)
Te(2)#21-Cu(1)-Cu(1)#5	139.9(4)	138.1(4)	141.7(4)
Te(2)#21-Cu(1)-Cu(2)	72.8(3)	68.5(3)	77.2(3)
Te(2)#19-Cu(1)-Te(3)	97.6(2)	96.7(2)	98.5(2)
Te(2)#19-Cu(1)-Cu(1)#5	69.1(2)	67.6(2)	70.6(3)
Te(2)#19-Cu(1)-Cu(2)	153.3(4)	151.3(4)	155.5(4)
Te(3)-Cu(1)-Cu(1)#5	102.8(3)	101.8(3)	103.7(3)
Te(3)-Cu(1)-Cu(2)	71.6(3)	68.0(3)	75.3(3)
Cu(1)#5-Cu(1)-Cu(2)	136.1(5)	131.8(5)	140.3(4)
La(1)#21-Cu(2)-La(1)#18	108.57(5)	107.39(5)	109.78(5)
La(1)#21-Cu(2)-Te(1)#20	67.54(4)	65.87(4)	69.20(4)
La(1)#21-Cu(2)-Te(1)#18	63.36(4)	62.30(4)	64.43(4)
La(1)#21-Cu(2)-Te(2)#21	63.23(4)	61.86(4)	64.64(4)
La(1)#21-Cu(2)-Te(3)	159.55(7)	156.17(7)	162.78(7)
La(1)#21-Cu(2)-Cu(1)	118.9(3)	116.3(3)	121.6(3)
La(1)#21-Cu(2)-Cu(2)#15	67.22(6)	65.32(6)	69.20(6)
La(1)#18-Cu(2)-Te(1)#20	65.97(4)	63.42(4)	68.50(4)
La(1)#18-Cu(2)-Te(1)#18	67.86(4)	66.99(4)	68.83(4)
La(1)#18-Cu(2)-Te(2)#21	166.43(8)	164.68(7)	168.23(7)
La(1)#18-Cu(2)-Te(3)	69.69(5)	66.35(4)	73.05(5)
La(1)#18-Cu(2)-Cu(1)	94.6(3)	87.7(3)	101.6(3)
La(1)#18-Cu(2)-Cu(2)#15	125.96(8)	124.43(7)	127.61(8)
Te(1)#20-Cu(2)-Te(1)#18	92.40(5)	90.65(5)	93.98(5)
Te(1)#20-Cu(2)-Te(2)#21	100.74(7)	99.69(6)	101.69(7)
Te(1)#20-Cu(2)-Te(3)	125.65(7)	120.13(7)	131.19(8)
Te(1)#20-Cu(2)-Cu(1)	72.9(3)	70.9(3)	74.8(3)
Te(1)#20-Cu(2)-Cu(2)#15	134.45(9)	131.56(9)	137.27(9)
Te(1)#18-Cu(2)-Te(2)#21	113.70(7)	109.52(6)	118.04(7)
Te(1)#18-Cu(2)-Te(3)	98.60(6)	97.23(6)	100.16(6)
Te(1)#18-Cu(2)-Cu(1)	160.9(3)	154.1(3)	167.2(3)
Te(1)#18-Cu(2)-Cu(2)#15	62.84(6)	61.08(5)	64.63(6)
Te(2)#21-Cu(2)-Te(3)	121.73(7)	119.23(7)	124.10(7)
Te(2)#21-Cu(2)-Cu(1)	81.6(3)	78.3(3)	84.6(3)
Te(2)#21-Cu(2)-Cu(2)#15	62.25(6)	58.61(6)	66.07(6)
Te(3)-Cu(2)-Cu(1)	81.0(3)	76.6(3)	85.3(4)

Te(3)-Cu(2)-Cu(2)#15	96.87(8)	93.92(8)	99.85(8)
Cu(1)-Cu(2)-Cu(2)#15	136.1(3)	131.1(3)	141.0(3)

Symmetry transformations used to generate equivalent atoms:
(1) x1,x2+1,x3,x4 (2) -x1+2,x2+1/2,-x3,-x3+x4+1/2 (3) -x1+1,x2+1/2,-x3,-x3+x4+1/2 (4) -x1+1,-x2+1,-x3,-x4 (5) x1,-x2+1/2,x3,x3-x4+1/2 (6) x1+1,x2+1,x3,x4 (7) x1+1,-x2+1/2,x3,x3-x4+1/2 (8) x1,x2-1,x3,x4 (9) -x1+2,x2-1/2,-x3+1,-x3+x4+1/2 (10) -x1+1,x2+1/2,-x3+1,-x3+x4+1/2 (11) -x1+1,-x2,-x3+1,-x4 (12) x1+1,x2,x3,x4 (13) -x1+1,-x2,-x3,-x4 (14) -x1+1,-x2+1,-x3+1,-x4 (15) x1,-x2-1/2,x3,x3-x4+1/2 (16) -x1+2,x2-1/2,-x3,-x3+x4+1/2 (17) -x1+2,x2+1/2,-x3+1,-x3+x4+1/2 (18) -x1+1,x2-1/2,-x3,-x3+x4+1/2 (19) -x1+1,x2-1/2,-x3+1,-x3+x4+1/2 (20) x1-1,x2,x3,x4 (21) x1-1,x2-1,x3,x4

REFERENCES

(1) Toby, B. H.; Von Dreele, R. B. GSAS-II: the genesis of a modern open-source all purpose crystallography software package. *Applied Crystallography* **2013**, *46* (2), 544-549.
(2) Sheldrick, G. M. SHELXT–Integrated space-group and crystal-structure determination. *Foundations of Crystallography* **2015**, *71* (1), 3-8.
(3) Sheldrick, G. M. A short history of SHELX. *Foundations of Crystallography* **2008**, *64* (1), 112-122.
(4) Dolomanov, O. V.; Bourhis, L. J.; Gildea, R. J.; Howard, J. A.; Puschmann, H. OLEX2: a complete structure solution, refinement and analysis program. *Applied Crystallography* **2009**, *42* (2), 339-341.
(5) Petříček, V.; Dušek, M.; Palatinus, L. Crystallographic computing system JANA2006: general features. *Zeitschrift für Kristallographie-Crystalline Materials* **2014**, *229* (5), 345-352.
(6) Palatinus, L.; Chapuis, G. SUPERFLIP–a computer program for the solution of crystal structures by charge flipping in arbitrary dimensions. *Applied Crystallography* **2007**, *40* (4), 786-790.
(7) Farrow, C.; Juhas, P.; Liu, J.; Bryndin, D.; Božin, E.; Bloch, J.; Proffen, T.; Billinge, S. PDFfit2 and PDFgui: computer programs for studying nanostructure in crystals. *Journal of Physics: Condensed Matter* **2007**, *19* (33), 335219.
(8) Prescher, C.; Prakapenka, V. B. DIOPTAS: a program for reduction of two-dimensional X-ray diffraction data and data exploration. *High Pressure Research* **2015**, *35* (3), 223-230.
(9) Le Bail, A.; Duroy, H.; Fourquet, J. L. Ab-initio structure determination of $LiSbWO_6$ by X-ray powder diffraction. *Materials Research Bulletin* **1988**, *23* (3), 447-452.
(10) Vinet, P.; Rose, J. H.; Ferrante, J.; Smith, J. R. Universal features of the equation of state of solids. *Journal of Physics: Condensed Matter* **1989**, *1* (11), 1941-1963.
(11) Angel, R. J.; Alvaro, M.; Gonzalez-Platas, J. EosFit7c and a Fortran module (library) for equation of state calculations. *Zeitschrift für Kristallographie-Crystalline Materials* **2014**, *229* (5), 405-419.
(12) Gonzalez-Platas, J.; Alvaro, M.; Nestola, F.; Angel, R. EosFit7-GUI: a new graphical user interface for equation of state calculations, analyses and teaching. *Applied Crystallography* **2016**, *49* (4), 1377-1382.
(13) Wang, Y.; Zhao, H.; Shahabfar, S.; Ie, T. S.; Sasmal, S.; Rosenkranz, S.; Wolverton, C.; Chung, D. Y.; Kanatzidis, M. G. La_3CuTe_5: A Narrow-Gap Semiconductor with Indirect Gap and Dual-Regime Thermally Activated Transport. *Inorganic Chemistry* **2025**, *64* (42), 20977-20985.

(14) Kresse, G.; Furthmüller, J. Efficiency of ab-initio total energy calculations for metals and semiconductors using a plane-wave basis set. *Computational Materials Science* **1996**, *6* (1), 15-50.
(15) Perdew, J. P.; Burke, K.; Ernzerhof, M. Generalized gradient approximation made simple. *Physical Review Letters* **1996**, *77* (18), 3865.
(16) Kirklin, S.; Saal, J. E.; Meredig, B.; Thompson, A.; Doak, J. W.; Aykol, M.; Rühl, S.; Wolverton, C. The Open Quantum Materials Database (OQMD): assessing the accuracy of DFT formation energies. *npj Computational Materials* **2015**, *1* (1), 15010.
(17) Togo, A.; Oba, F.; Tanaka, I. First-principles calculations of the ferroelastic transition between rutile-type and $CaCl_2$-type SiO_2 at high pressures. *Physical Review B* **2008**, *78* (13), 134106.
